# Supplementary material for: Microtubule-Actomyosin Mechanical Cooperation during Contact Guidance Sensing
Source: Cell Rep. Author manuscript; Available in PMC 2018 Nov 9. (PMC6226003; doi:10.1016/j.celrep.2018.09.030)
Supplement: 1 [file NIHMS1510161-supplement-1.pdf]

Cell Reports, Volume *25*

Supplemental Information

Microtubule-Actomyosin Mechanical Cooperation during Contact Guidance Sensing

Erdem D. Tabdanov, Vikram Puram, Alexander Zhovmer, and Paolo P. Provenzano

Supplemental Figures:

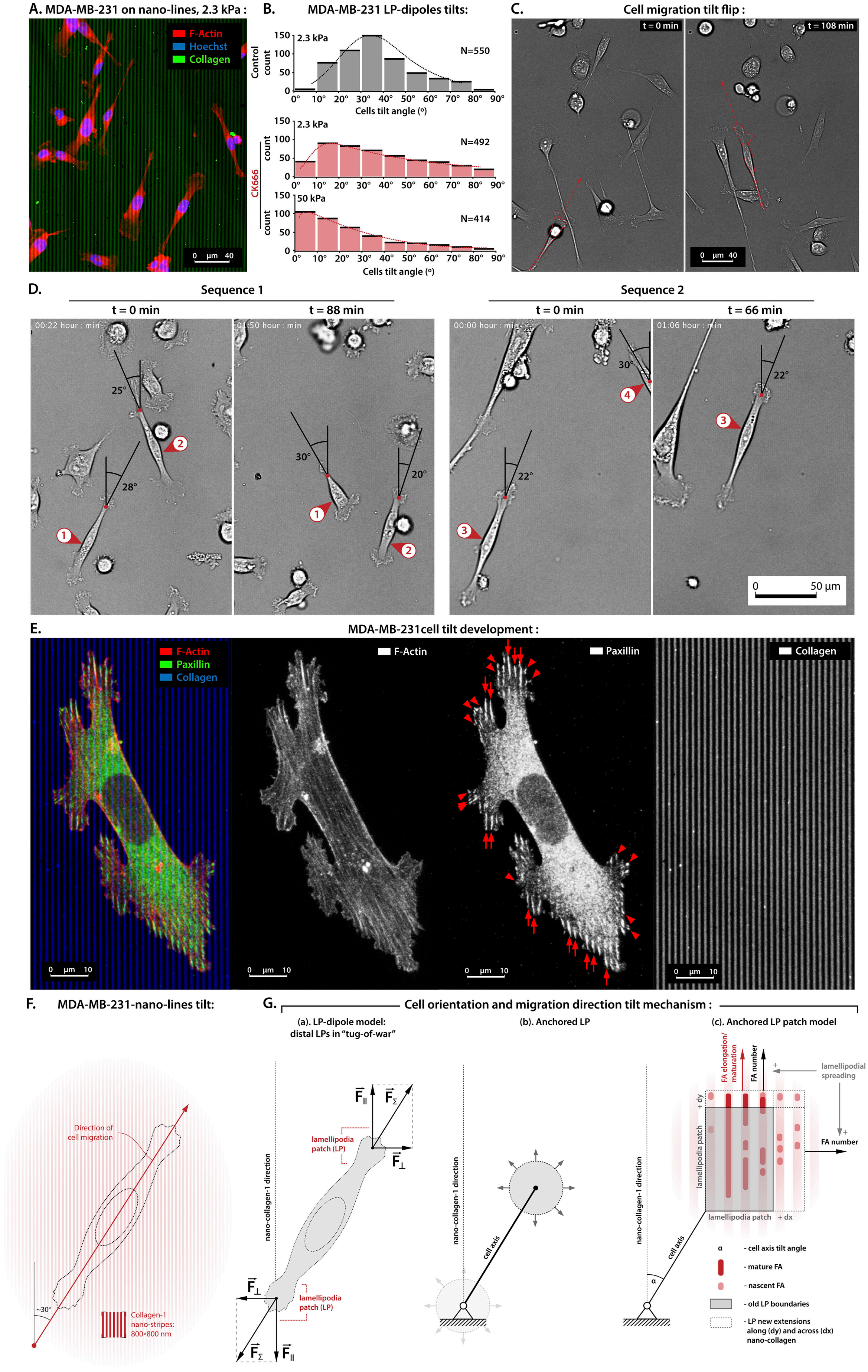

Figure S1. Related to Figures 2 and 3. Carcinoma cell axis and migration tilt relative to collagen nanoline "fibers" on compliant 2.3 kPa substrates
(A) Overview of tilting of cell populations on flat soft collagen nanolines. **(B)** Distribution of cell tilts on nanolines for the control case (+*DMSO*, 2.3kPa) and during Arp2/3 inhibition (+*CK666*, 2.3 and 50kPa collagen nanolines). Analysis of orientation of rod-shaped cells on the compliant nanolines shows the populational tilt amassing at an ~30º angle. **(C)** Time sequence of tilted cell migration capturing conservation of cell tilt during a change in cell migration direction (red outlining contour). Analysis of live cell migration dynamics indicates that the tilt is dynamically steady (see also Movie 2) and that MDA-MB-231 cells show conservation of migration tilt angle of ~30º irrespectively of of the choice between all 4 possible quadrants of cell migration directions (i.e. the migration flip or "reflection"). **(D)** Change in cell tilt tracked across two video sequences. Tilts are indicated with angles values (*black*), each flipping cell is marked with a number (*red*). **(E)** Early (t<30 min) cell elongation into LP-dipoles and its tilt (G'=2.3kPa): elongating mature FA - *red arrows*, newly established FAs - *red arrowheads*. **(F)** Schematic of a linearized cell tilted relative to the collagen nanolines. **(G)** (a) - Tilting LP-dipole cell with both $F_{||}$ - vertical forces, $F_{\perp}$ - transverse forces. (b) - LP-dipole represented as an anchored LP, (c) - Detailed schematic of LP migration tilt formation: resultant cell orientation emerges from superposition of different forces vector direction that are generated by FA maturation and elongation along the collagen nanolines, and across collagen nanolines stripes with increasing numbers of anchoring sites - nascent FAs. Thus, from this data we argue that MT-induced cell linearization into LP-dipoles on flat compliant nanolines (see Figure 2) links contractile and protrusive lamellipodial dynamics at the cell distal regions, enabling cells' slightly angled orientation along quasi-2D collagen nanolines. That is, structural and mechanical analysis of distal lamellipodii indicates a radial distribution of both FAs (Figure 2 and S2) and traction forces (Figure S2) in LPs that behave as semi-autonomous elements, but are linked via the MT-rich cell body. Thus, the rod-shaped cell phenotype represent a configuration with active dual distal LPs, interconnected into a single system (i.e. an LP-dipole entangled into a "tug-of-war" configuration that creates symmetry and principally differs from the single lamellipodia phenotype that results in more circular traction symmetry (Figure S2). Indeed, since protrusion of individual LP is primarily guided by maturation of FAs along (||) the continuous length of the CG cues and since new nascent FAs (per single lamellipodial undulation) are established as the membrane protrudes perpendicular to the nanolines (⊥), the balance of mature aligned FAs and nascent FAs protrusion establishes LP-dipole directionality that is tilted relative to the nanolines lines (panel F). Lastly, the model proposed here predicts that suppression of lamellipodial dynamics would decrease the rate and area at which the nascent FAs are established, shifting cell spreading toward the direction of mature focal adhesion elongation, hence, decreasing cell tilts. Indeed, Arp2/3 inhibition results in a decrease in lamellipodial structures at the cell ends and a significant decrease in tilt angle from ~30º to ~5-10º on both compliant and stiff nanoline (Figure 2A and S2), supporting our model. Further, analysis of the traction forces in the Arp2/3-inhibited rod-shaped cells that are devoid of LP-generated off-axis forces indicates increased force alignment along the contact guidance cues (Figure S2).

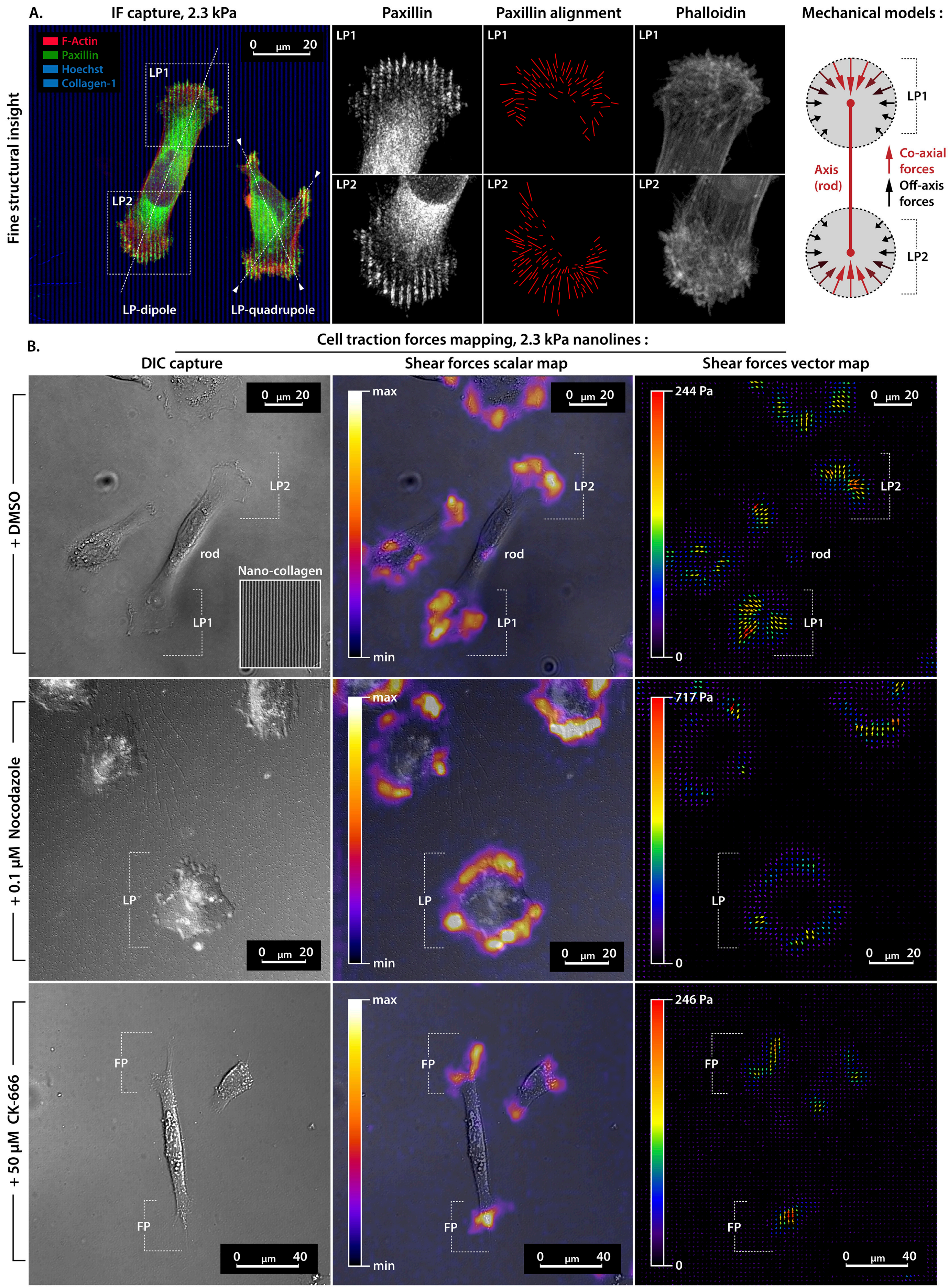

A.
IF capture, 2.3 kPa
Paxillin
Paxillin alignment
Phalloidin
Mechanical models :
Fine structural insight
F-Actin
Paxillin
Hoechst
Collagen-1
0 μm 20
LP1
LP2
LP-dipole
LP-quadrupole
Co-axial forces
Off-axis forces
Axis (rod)
B.
Cell traction forces mapping, 2.3 kPa nanolines :
DIC capture
Shear forces scalar map
Shear forces vector map
+ DMSO
rod
Nano-collagen
max
min
244 Pa
0
+ 0.1 μM Nocodazole
LP
717 Pa
+ 50 μM CK-666
FP
246 Pa
0 μm 40

Figure S2. Related to Figures 2 and 3. Analysis of cytoskeleton structure and stress alignments within distal lamellipodial patches (LP) in LP-dipoles on compliant 2.3kPa collagen nanolines

(A) Overview of cells on compliant (G'=2.3kPa) collagen nanolines substrates (*left panel*). Boxed areas with lamellipodia patches (LP1 and LP2) are magnified for detailed view (*right panels*). Note the paxillin puncta alignment (red) forms a radially organized pattern within each distal LP, outlining the partial traction autonomy of each distal LP. Simplified rod-shaped cell schematics outlines distal LPs interlinked with elongated cell body (rod). **(B)** Traction force microscopy on compliant nanolines showing the spatial distribution of traction stresses in control cells (+*DMSO*), circular cells with singular lamellipodia (+*Nocodazole*) and in rod-shaped cells with disrupted lamellipodial dynamics (+*CK666*). Note, inward-directed radially arrayed stresses in distal LPs in control rod-shaped cells (+*DMSO*). Thus, while the principal of the cell has co-axial forces communicating along the principal axis of the cell from LP1 to LP2 in an end-to-end manner, radial off-axis stresses suggest autonomous LP inward-directed contractility within each individual distal LP. MTs disruption-induced single circular lamellipodia (+*Nocodazole*) shows inward-directed peripheral stresses. Arp2/3 inhibition induces predominantly co-axial principal stresses, conforming to the observed protrusions.

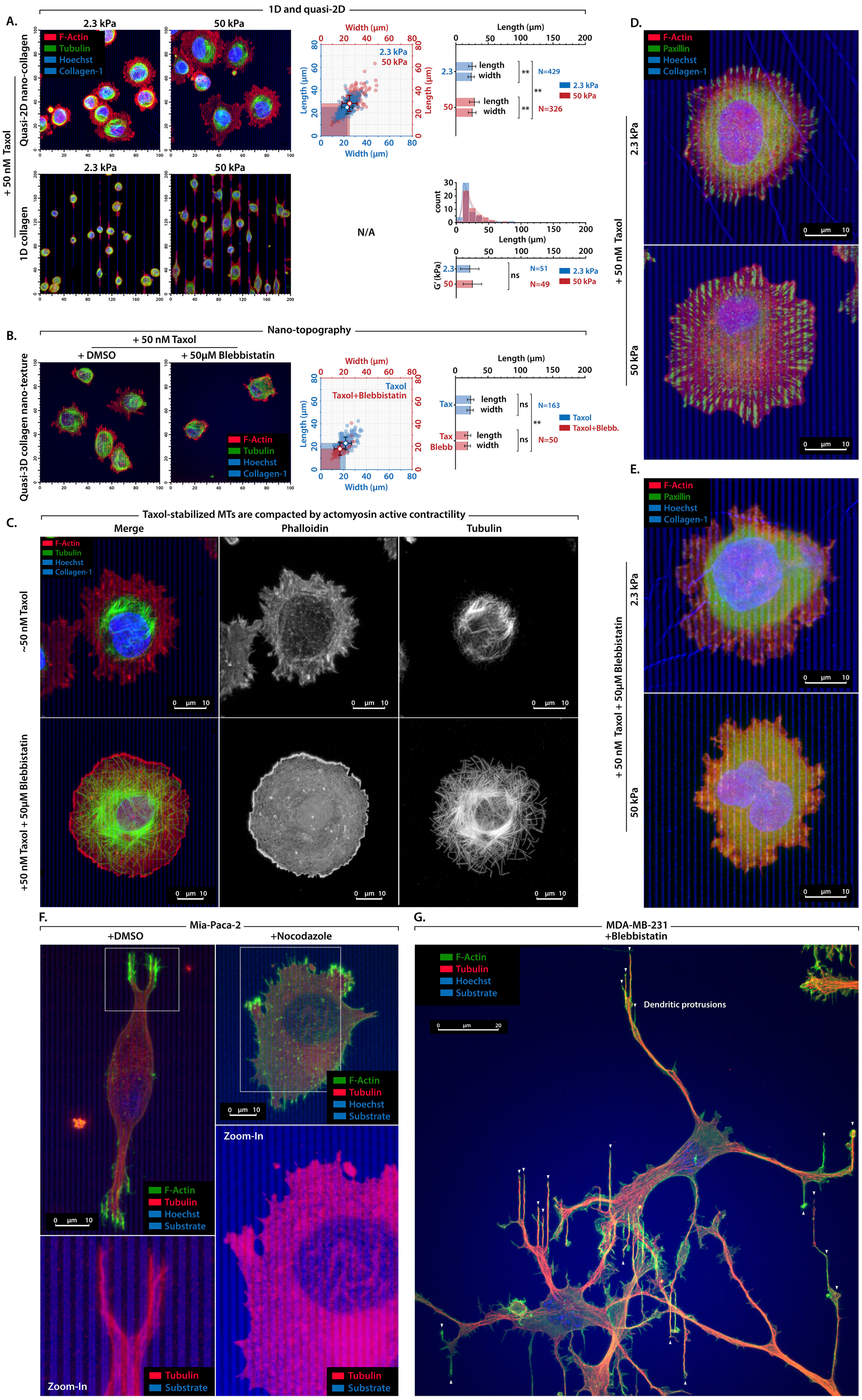

1D and quasi-2D
A.
2.3 kPa
50 kPa
Quasi-2D nano-collagen
1D collagen
+ 50 nM Taxol
F-Actin
Tubulin
Hoechst
Collagen-1
Width (μm)
Length (μm)
2.3 kPa
50 kPa
length
width
N=429
N=326
N/A
count
G' (kPa)
N=51
N=49
ns
B.
Nano-topography
+ 50 nM Taxol
+ DMSO
+ 50μM Blebbistatin
Quasi-3D collagen nano-texture
Taxol
Taxol+Blebbistatin
Tax
Tax Blebb
N=163
N=50
Taxol+Blebb.
C.
Taxol-stabilized MTs are compacted by actomyosin active contractility
Merge
Phalloidin
Tubulin
~50 nM Taxol
+50 nM Taxol + 50μM Blebbistatin
D.
Paxillin
+ 50 nM Taxol
E.
+ 50 nM Taxol + 50μM Blebbistatin
F.
Mia-Paca-2
+DMSO
+Nocodazole
Substrate
Zoom-In
G.
MDA-MB-231
+Blebbistatin
Dendritic protrusions

Figure S3. Related to Figures 2-5. (A-E) Taxol treatment effects on MDA-MB-231 cell alignment in response to 1D, quasi-2D, and nano-textured topographic collagen guidance cues. (F) Pancreatic ductal adenocarcinoma response to topographic collagen CG cues. (G) Blebbistatin-induced dendritic protrusions guidance by collagen nano-topographic CG cues

(A) Taxol-treated MDA-MB-231 cell elongation and spreading on compliant and stiff collagen nanolines (quasi-2D) and along 1D collagen microlines. Cell spreading widths and lengths (when applicable) are shown on the right panels. **(B)** Taxol-treated MDA-MB-231 cell spreading and elongation along and across nanotextured collagen surfaces. Cell spreading width and lengths are shown on the right panels. **(C)** Actomyosin-dependent spatial redistribution of taxol-stabilized microtubules in cells on compliant (G'=2.3kPa) nanolines. Taxol-treated MDA-MB-231 cells on compliant quasi-2D nano-collagen surfaces demonstrate isotropic (i.e. circular) spreading. In contractile cells, taxol-stabilized MTs are compacted around the nucleus while low-contractile blebbistatin-treated cells display scattered MTs distribution. **(D and E)** FA staining (paxillin) in cells on compliant (2.3kPa) and stiff (50kPa) nanolines in Taxol and Taxol+blebbistatin conditions. **(F)** Mia-Paca-2 pancreatic adenocarcinoma cell alignment to collagen nanotextures. Cells in control conditions (+*DMSO*) feature robust alignment and MT conformity to the underlying nanogrooves, consistent with findings in MDA-MB-231 breast carcinoma cells. Nocodazole-induced MT disruption disables cell alignment to the nanotexture. **(G)** Blebbistatin-induced dendritic protrusions in MDA-MB-231 cells display a partial conformity and alignment to the underlying nanogrooved textures with small MT-rich in-groove protrusions (*white arrowheads*). Data in right panels are mean ±s.d.; ns indicates no significant difference between groups; *p<0.05, **p<0.001.

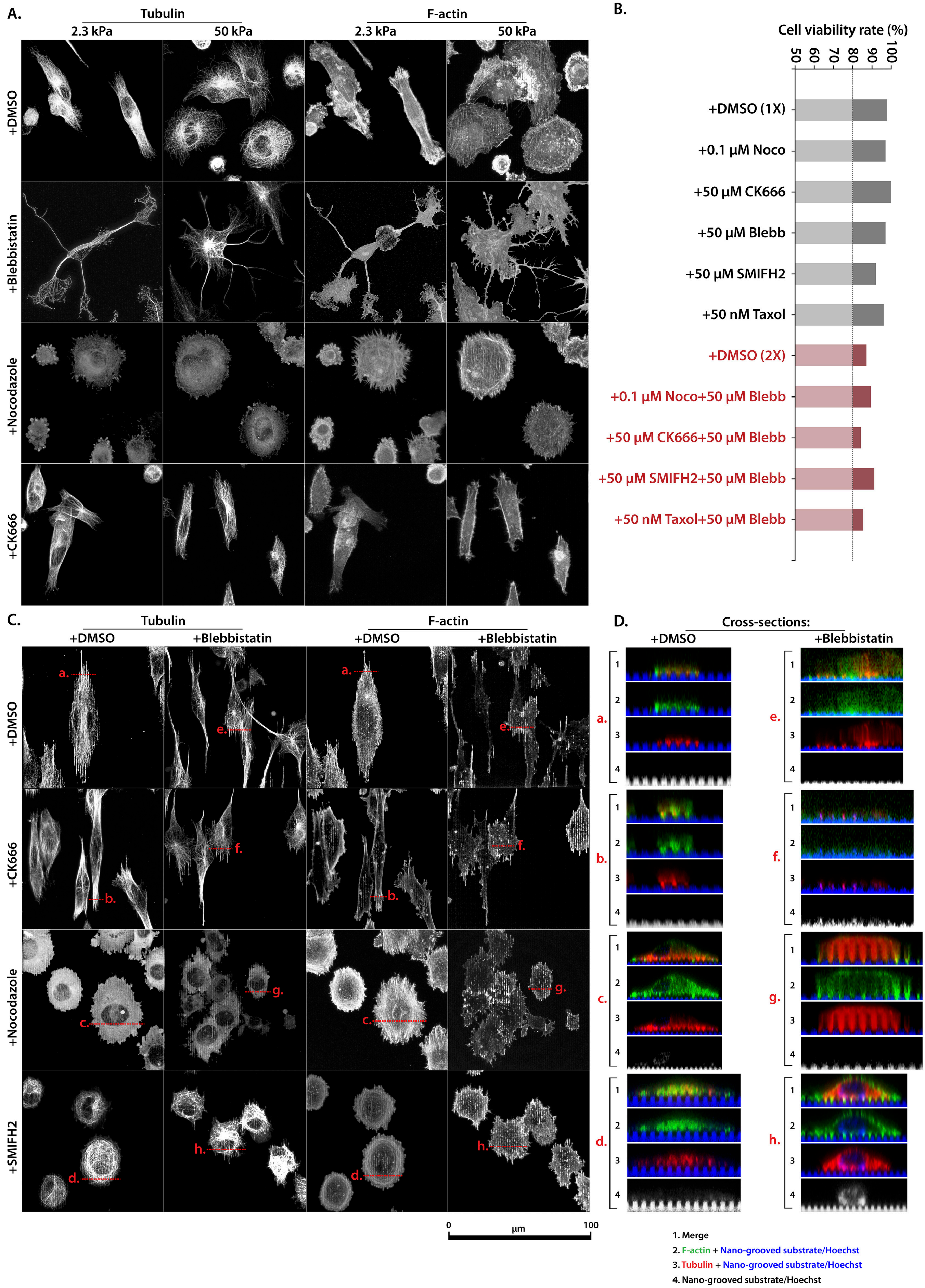
A.
Tubulin
F-actin
2.3 kPa
50 kPa
+DMSO
+Blebbistatin
+Nocodazole
+CK666
B.
Cell viability rate (%)
50
60
70
80
90
100
+DMSO (1X)
+0.1 µM Noco
+50 µM CK666
+50 µM Blebb
+50 µM SMIFH2
+50 nM Taxol
+DMSO (2X)
+0.1 µM Noco+50 µM Blebb
+50 µM CK666+50 µM Blebb
+50 µM SMIFH2+50 µM Blebb
+50 nM Taxol+50 µM Blebb
C.
Tubulin
F-actin
+DMSO
+Blebbistatin
+DMSO
+CK666
+Nocodazole
+SMIFH2
a.
b.
c.
d.
e.
f.
g.
h.
0
µm
100
D.
Cross-sections:
+DMSO
+Blebbistatin
1
2
3
4
1. Merge
2. F-actin + Nano-grooved substrate/Hoechst
3. Tubulin + Nano-grooved substrate/Hoechst
4. Nano-grooved substrate/Hoechst

Figure S4. Related to Figures 3 and 5. (A) Tubulin and F-actin channels for cells on quasi-2D collagen nanolines depicted on Figure 3A. **(B)** Cell viability rates for pharmacological treatments outlined in Figures 3 and 5. **(C)** Tubulin and F-actin channels for cells on collagen nanotextured CG cues that are depicted in Figure 5A. **(D)** CG-transverse cross-sections of the cells depicted in Figure 5A and (C) (*corresponding cross-sections marked with red letters and dashed lines in panel C*).

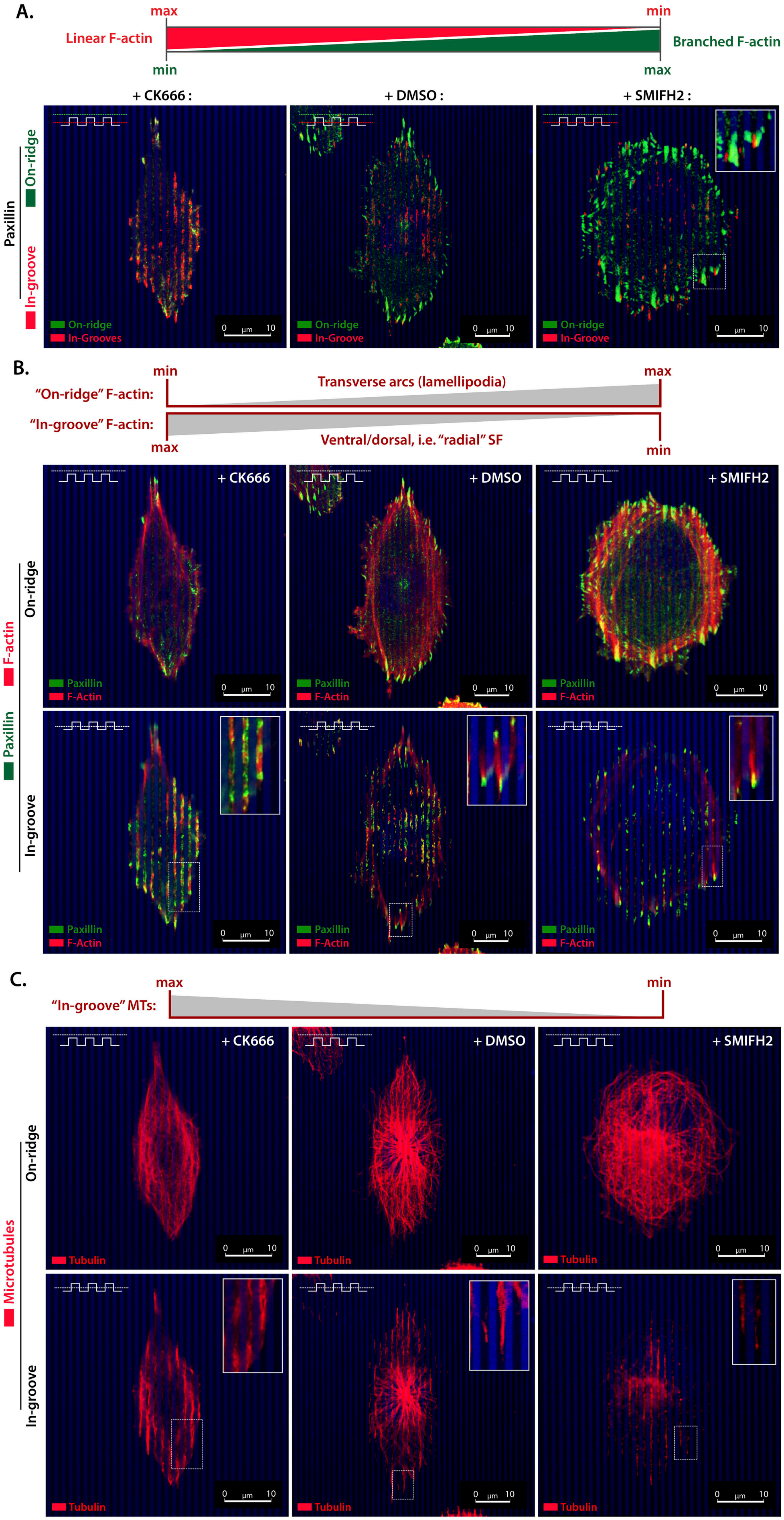
A.
max
min
Linear F-actin
Branched F-actin
min
max
+ CK666 :
+ DMSO :
+ SMIFH2 :
Paxillin
On-ridge
In-groove
On-ridge
In-Grooves
In-Groove
0 µm 10
B.
min
max
"On-ridge" F-actin:
Transverse arcs (lamellipodia)
"In-groove" F-actin:
Ventral/dorsal, i.e. "radial" SF
max
min
+ CK666
+ DMSO
+ SMIFH2
F-actin
Paxillin
On-ridge
In-groove
Paxillin
F-Actin
C.
max
min
"In-groove" MTs:
Microtubules
On-ridge
In-groove
Tubulin

Figure S5. Related to Figures 4 and 5. Apr2/3- and Formins-dependent mechanisms regulate Transverse Arcs and Ventral/Dorsal Stress Fiber F-actin architectures that regulate MT translocation between in-grooves and on-ridges layers during cellular sensing of “2.5D” collagen nanotextured CG cues.

(A) Translocation of FAs (*paxillin*) toward “in-groove” (*red*) or “on-ridge” (*green*) cell-CG interface layers during Arp2/3 (+*CK666*) or Formins (+*SMIFH2*) inhibitions, respectively. **(B)** Arp2/3 suppression (+*CK666*) diminishes on-ridge lamellipodial branched actin cytoskeleton, decreases transverse arcs, and increases robust in-groove linear F-actin structures, reminiscent to ventral/dorsal stress-fibers (*see zoomed “in-groove” inset*). Formins inhibition (+*SMIFH2*) shifts the actin architecture toward on-ridge transverse arcs and suppresses SF linear F-actin in the in-groove layer, inducing prominent F-actin translocation into on-ridge lamellipodial layer that spans atop and across multiple nanoridges. Control cells (+*DMSO*) feature both robust in-groove and on-ridge cytoskeleton architectures, representing a structural balance between Arp2/3- and Formins-regulated TA versus SF actin architectures. **(C)** Visualization of the corresponding on-ridge and in-groove MTs architectures in the cells under treatments outlined in (A) and (B). Note, decrease of the in-groove MTs as cellular F-actin transitions from predominantly linear (+*CK666*) through intermediate (+*DMSO*) to the branched, lamellipodial form (+*SMIFH2*). The measured percentage of cell-nanotexture interface areas featuring MT-positive invasive in-grooves dents for all three cases are shown on Figure 5E.
