# Supplementary material for: Microtubule-Actomyosin Mechanical Cooperation during Contact Guidance Sensing
Source: Cell Rep. Author manuscript; Available in PMC 2018 Nov 9. (PMC6226003; doi:10.1016/j.celrep.2018.09.030)
Supplement: 7 [file NIHMS1510161-supplement-7.pdf]

Cell Reports

Report

Microtubule-Actomyosin Mechanical Cooperation during Contact Guidance Sensing

Graphical Abstract

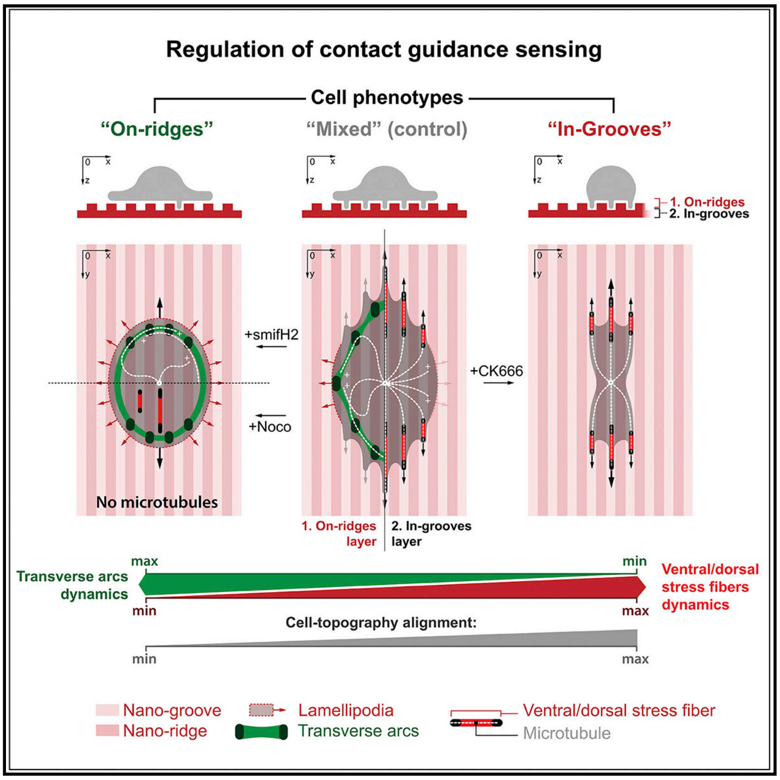

Highlights

- Lamellipodia and microtubule dynamics regulate contact guidance from flat nanolines
- The microtubule network acts as an intracellular mechano-structural scaffold
- Microtubule scaffolds regulate steric interactions with nanotopographic guidance cues
- Arp2/3 and Formins-dependent actin architectures compete to regulate contact guidance

Authors

Erdem D. Tabdanov, Vikram Puram, Alexander Zhovmer, Paolo P. Provenzano

Correspondence

etabdano@umn.edu (E.D.T.),
pprovenz@umn.edu (P.P.P.)

In Brief

Aligned extracellular matrix architectures in tumors direct migration of invasive cancer cells. Tabdanov et al. show that the mechanical properties of aligned extracellular matrix environments influence invasive cell behavior and define a mechanical role for microtubules and actomyosin-microtubule interactions during sensing of contact guidance cues that arise from aligned extracellular matrix.

Tabdanov et al., 2018, Cell Reports *25*, 328–338
October 9, 2018 © 2018 The Author(s).
https://doi.org/10.1016/j.celrep.2018.09.030

CellPress

OPEN ACCESS
CellPress

Cell Reports
Report

Microtubule-Actomyosin Mechanical Cooperation during Contact Guidance Sensing

Erdem D. Tabdanov,[1,2,*] Vikram Puram,[1,2,7] Alexander Zhovmer,[3,7] and Paolo P. Provenzano[1,2,4,5,6,8,*]
[1]Department of Biomedical Engineering, University of Minnesota, Minneapolis, MN 55455, USA
[2]University of Minnesota Physical Sciences in Oncology Center, Minneapolis, MN 55455, USA
[3]NIH, Bethesda, MD 20892, USA
[4]Masonic Cancer Center, University of Minnesota, Minneapolis, MN 55455, USA
[5]Stem Cell Institute, University of Minnesota, Minneapolis, MN 55455, USA
[6]Institute for Engineering in Medicine, University of Minnesota, Minneapolis, MN 55455, USA
[7]These authors contributed equally
[8]Lead Contact
*Correspondence: etabdano@umn.edu (E.D.T.), pprovenz@umn.edu (P.P.P.)
https://doi.org/10.1016/j.celrep.2018.09.030

SUMMARY

Cancer cell migration through and away from tumors is driven in part by migration along aligned extracellular matrix, a process known as contact guidance (CG). To concurrently study the influence of architectural and mechanical regulators of CG sensing, we developed a set of CG platforms. Using flat and nanotextured substrates with variable architectures and stiffness, we show that CG sensing is regulated by substrate stiffness and define a mechanical role for microtubules and actomyosin-microtubule interactions during CG sensing. Furthermore, we show that Arp2/3-dependent lamellipodia dynamics can compete with aligned protrusions to diminish the CG response and define Arp2/3- and Formins-dependent actin architectures that regulate microtubule-dependent protrusions, which promote the CG response. Thus, our work represents a comprehensive examination of the physical mechanisms influencing CG sensing.

INTRODUCTION

Sensing contact guidance cues and subsequent directed cell migration are essential phenomena that govern numerous processes such as morphogenesis (Daley and Yamada, 2013), immune cell migration (Friedl and Bröcker, 2000), and metastatic dissemination (Conklin et al., 2011; Patsialou et al., 2013; Provenzano et al., 2006). However, despite progress toward understanding the principles of cell-extracellular matrix (ECM) architecture sensing, contradictory paradigms have emerged. For example, actomyosin contractility has been reported to be both dispensable or necessary for fibroblast contact guidance (CG) along one-dimensional (1D) cues (Doyle et al., 2009, 2012; Guetta-Terrier et al., 2015), while carcinoma cell contractility is essential for ECM alignment (Carey et al., 2013; Provenzano et al., 2008), but dispensable for migration through prealigned ECM (Provenzano et al., 2008). Thus, both cell and ECM mechanics may influence the 1D, 2D, or 3D CG response (Carey et al., 2015; Chang et al., 2013; Doyle et al., 2009; Provenzano et al., 2006, 2008; Ray et al., 2017). However, surprisingly opposite trends in CG behavior have been reported depending on whether traction is modulated intrinsically (by targeting myosin) or extrinsically (by changing substrate stiffness) (Nuhn et al., 2018). As such, questions remain regarding the influence of effective traction during CG sensing. Therefore, novel platforms are needed that allow for concurrent control of both mechanical rigidity and ECM architecture across multiple scales to parse out complex CG sensing behavior.

Regulation of CG-directed cell migration has been attributed to lamellipodia along protrusive edges, as well as filopodia, pseudopodia, and invadopodia (Albuschies and Vogel, 2013; Doyle et al., 2009, 2012; Jacquemet et al., 2015; Teixeira et al., 2003). In sum, resultant cell orientation can be attributed to competitive dynamics between multidirectional lamellipodia spreading featuring Arp2/3-branched F-actin with circular contractile transverse arcs and more directed protrusions featuring Formins-driven radially directed ventral and dorsal stress fibers (SFs) (Hotulainen and Lappalainen, 2006; Oakes et al., 2012), suggesting that concurrent counterbalancing cytoskeleton dynamics could regulate the robustness of the CG response, consistent with transverse lamellipodia spreading across densely arrayed lines that can compete with the directed CG response (Ramirez-San Juan et al., 2017; Romsey et al., 2014). A similar interference has also been suggested to influence CG along nanogrooves (Lee et al., 2016; Ray et al., 2017; Teixeira et al., 2003). However, the mechanisms governing cell conformity to CG topography are poorly understood. Intriguingly, reports relate microtubules (MTs) to topography sensing (Lee et al., 2016; Oakley and Brunette, 1995), cell conformity to fibrillar 3D network (Bouchet and Akhmanova, 2017; Rhee et al., 2007), and compression resistance in cell leading edge of contracting cells (Brangwynne et al., 2006), suggesting that increased understanding of the structural and mechanical roles of MTs during CG may increase our understanding of directed motility. Thus, here using engineered CG platforms, we address fundamental questions regarding competitive protrusion behavior and elucidate the physical and molecular mechanisms governing lamellipodia- and MT-regulated CG sensing.

 © 2018 The Author(s).
This is an open access article under the CC BY license (http://creativecommons.org/licenses/by/4.0/).

RESULTS

Engineering Multiscale Mechano-structural Contact Guidance Cues

The current paradigm of CG from 2D flat or textured surfaces links cell alignment (and directed migration) to alignment of focal adhesions (FAs), SFs, and directed cell protrusions (Doyle et al., 2009; Ramirez-San Juan et al., 2017; Ray et al., 2017; Romsey et al., 2014). However, the impact of mechanosensitivity during CG-directed cell alignment is far less explored due to challenges engineering environments with nanoscale and/or microscale CG cues of variable stiffness. As such, we designed platforms with type I collagen CG cues of defined mechanical rigidities and oriented architectures (i.e., dense quasi-2D nanolines, 1D microlines, and "2.5D" topographic CG cues: Figure 1; see STAR Methods for full platforms descriptions) to study CG sensing, and in particular, competitive dynamics between CG-directed protrusions versus non-oriented multidirectional spreading. Furthermore, the topographic features of nanotextured CG cues are sterically interactive at the nanoscale but can also allow multidirectional lamellipodial protrusions on the microscale (Ray et al., 2017), allowing us to capture mechanical and structural mechanisms of competition between distinct cell protrusion behaviors during CG sensing.

Lamellipodia and MT Dynamics Regulate Cell Alignment to Nanoline CG Cues

We first examined the relationship between FA and SF morphologies and cell alignment on compliant (2.3-kPa) and stiff (50-kPa) nanolines, and examined the roles of MTs and lamellipodial dynamics by pretreating cells with nocodazole (MT disruption) or CK666 (Arp2/3 inhibition), respectively. Control data (+DMSO) show that densely spaced nanolines induce multidirectional lamellipodia (Figure 2A). On compliant nanolines, cells undergo cell linearization into rod-shaped structures with distal lamellipodial patches (LPs) at the ends (i.e., an LP-dipole), where the contractile LP regions contain small FAs and poorly aligned F-actin in contrast to no detectable FAs and aligned F-actin in the rod regions (Figures 2A, 2C, and 2D; Video S1). Note that the cell tilt likely results from competitive dynamics between more random nascent FAs and more aligned mature FA (Figure S1; Video S2). Alternatively, stiff nanolines induce circular lamellipodia around each cell yet possess robust FAs and aligned F-actin along the CG cues (Figures 2A, 2C, and 2D; Video S3), suggesting that regional contractile cytoskeleton alignment is not the sole determining factor that governs cell alignment to CG architectures.

On both compliant and stiff nanolines, SFs and FAs in MT-disrupted cells remain predominantly co-aligned to nanolines and retain significantly larger FAs on stiffer nanolines (Figures 2A, 2C, and 2D). However, nocodazole-treated cells largely respond the same to both compliant and stiff nanolines, where cells on soft CG cues now behave similar to cells on stiff cues (Figure 2A). Indeed, morphology averaging reveals circular spreading across all three cases of linearization loss (Figure 2B), despite alignment of mature FAs and SFs. Interestingly, MT stabilization with Taxol also produces a circular cell morphology, with mature FAs, for both stiffnesses (Figure S3A), suggesting that inactive, stabilized, MTs are also insufficient for cell linearization. Furthermore, suppression of actomyosin contractility induces a redistribution of Taxol-stabilized MTs without significantly changing cell morphology, pointing toward physical interactions between contractile actomyosin and MT networks (Gardel et al., 2008; Picone et al., 2010). Thus, we conclude that MTs are required for guidance on soft nanolines and that, without proper MTs dynamics and MT-actin interactions, a mechanism of multidirectional lamellipodia protrusion drives strong cell circularities irrespective of FA and SF alignments.

In contrast to cell behavior after altering MTs, suppression of lamellipodia through inhibition of the Arp2/3 complex transformed cells into the rod phenotype, but with distal microspiked structures featuring small FAs instead of LPs (thus precluding actin analysis in lamellipodia regions), on both compliant and stiff nanolines (Figure 2A). Thus, our data suggest that MTs regulate single-cell axis alignment and that flat, densely spaced, nanolines allow protrusions to distribute across multiple nanolines, in contrast to more directed protrusions on nanotextures (Ray et al., 2017), with cells undergoing robust multidirectional lamellipodia-driven spreading, even when FA and actin are predominantly aligned to the nanolines, revealing a competing balance between CG-directed and multidirectional cell spreading.

The MT Network Acts as an Intracellular Mechano-structural Scaffold

Previous studies highlight potential structural (Picone et al., 2010; Rhee et al., 2007) and, to date, largely hypothesized mechanical (Lee et al., 2015; Rhee et al., 2007) roles of MTs during protrusion alignment to anisotropic ECMs. To further explore the MT-actin-myosin relationship, we perturbed MTs in concert with myosin-regulated traction forces or F-actin branching, without observing any adverse effects on cell viability (Figures 3, S3, and S4). In all linearization cases (+DMSO, 2.3 kPa; +CK666, 2.3 and 50 kPa), dense parallelized MT-bundles are a core structural element in the rod regions, in contrast to stiff control conditions (+DMSO, 50 kPa) that result in a circular cell phenotype with isotropic radially dispersed MTs (Figures 3A–3C and S3). Likewise, MT disruption with nocodazole results in loss of cell linearization in favor of predominantly circular cells (Figures 3A and 3B). Notably, findings that MT bundling within contractile actomyosin networks can lead to mechanical rigidification of sarcomeric structures (Robison et al., 2016), axons (Burnette et al., 2008), and cell protrusions (Bouchet and Akhmanova, 2017), and reported MT-actomyosin interactions (Dugina et al., 2016), are in agreement with our observed decompaction of Taxol-stabilized MTs after contractility inhibition (Figure S3C). Thus, in this context, we suggest that actomyosin-dependent MT parallelization and bundling results in a rigid "rod" section that mechanically separate, yet link, distal LPs into a symmetric "tug-of-war" configuration, influencing the CG response. We confronted this hypothesis by inhibiting myosin II-driven contractility (+Blebb), and we observe MT-actomyosin unbundling into incoherent, MT-positive, dendritic protrusions that are not aligned to CG cues (Figures 3A and 3B). Likewise, switching F-actin structure from the predominantly ventral and dorsal and/or ventral SF phenotype (termed dorsal and/or ventral SF here) to a predominantly transverse arcs phenotype (termed transverse arcs here) through inhibition of Formins results in loss of MT bundling in favor of a more

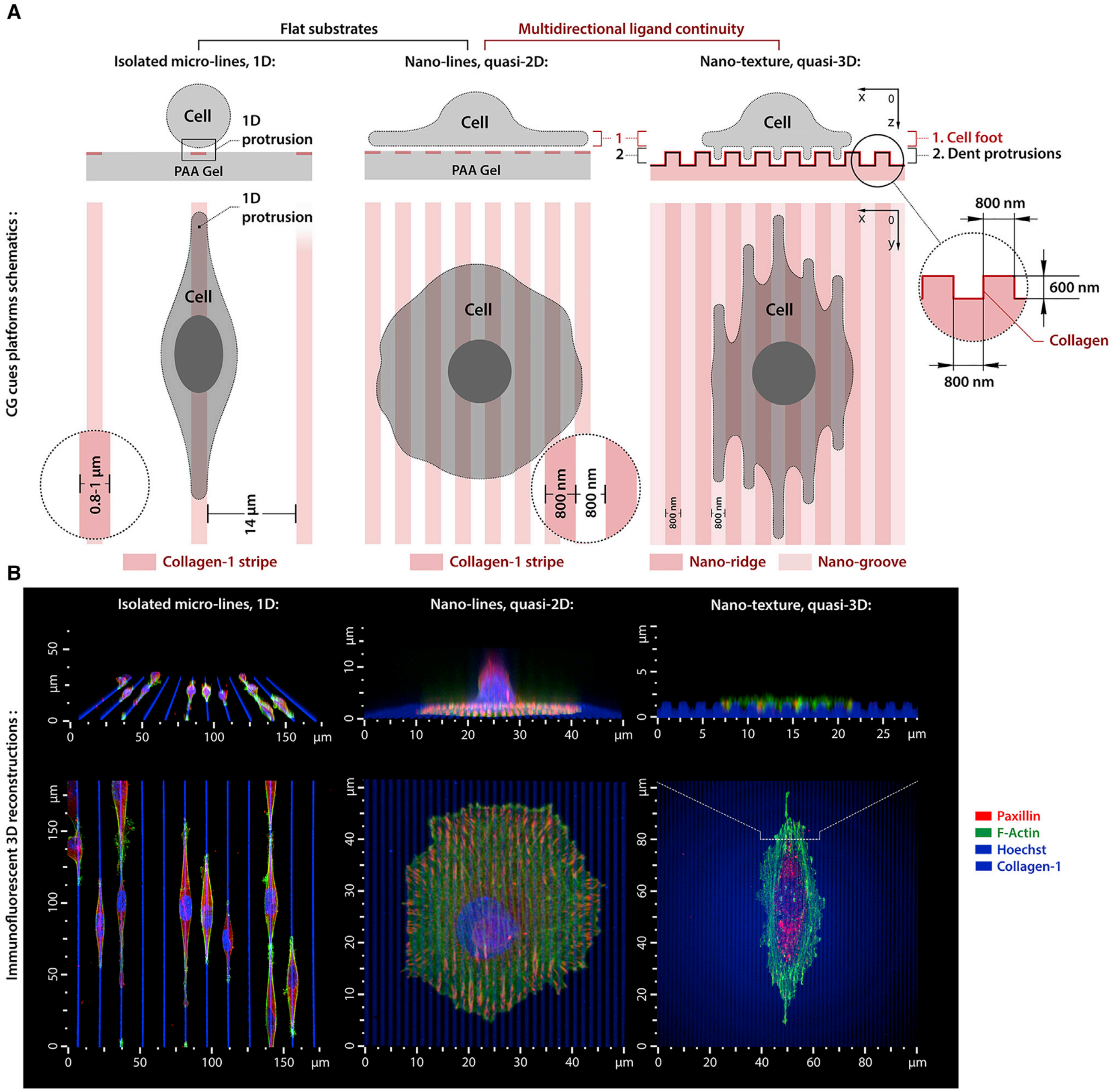

C

	Isolated micro-lines, 1D:	Nano-lines, quasi-2D:	Nano-texture, quasi-3D:
G′=2.3 kPa :	+	+	+
G′=50 kPa :	+	+	+
G′>>50 kPa :	-	-	+

Figure 1. Engineered Platforms to Induce Distinct Contact Guidance Responses

(A and B) Schematic (A) and 3D microscopy views (B) of MDA-MB-231 cell alignment in response to 1D (sparse type I collagen microlines), quasi-2D (dense collagen nanolines), and topographic (nanotextured collagen) CG cues.

(C) Shear moduli of the CG mechanical platforms.

random MT network constrained within the transverse arcs (Figure 3C). Thus, these collective findings suggest that MT networks serve as an intracellular mechanical scaffold that can mechanically compete for actomyosin contractile energy. As such, when FA-SF tractions are understimulated due to relatively soft nanolines, the actomyosin cytoskeleton can collapse onto the

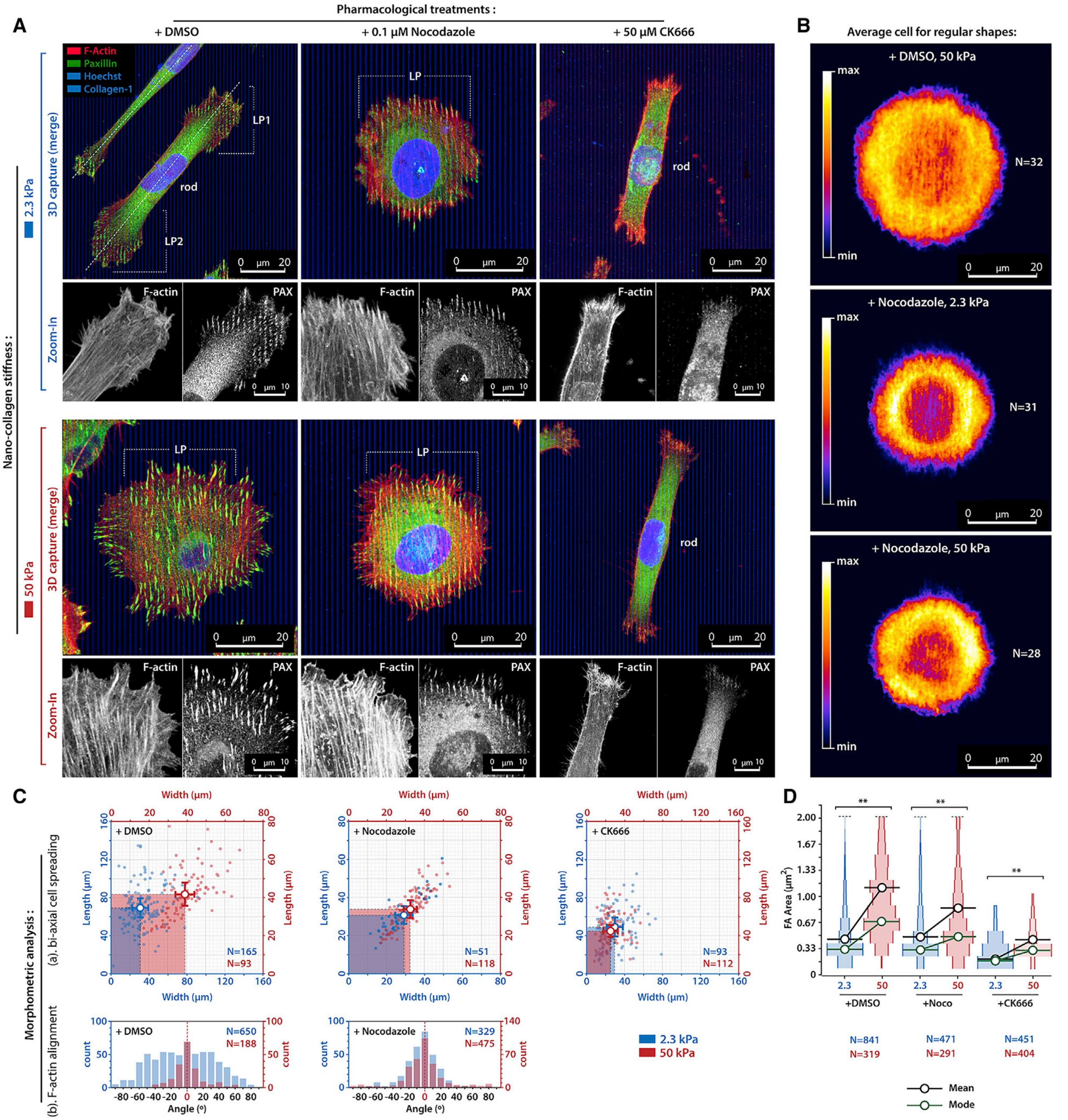

Figure 2. Mechano-regulated Cell Alignment to Collagen Nanoline CG Cues Is Regulated by Intact Microtubules and Arp2/3.

(A) MDA-MB-231 cells on compliant (2.3-kPa, top row) and stiff (50-kPa, bottom row) collagen nanoline substrates in control conditions (+DMSO), with disrupted MTs (+Nocodazole), and during Arp2/3 inhibition (+CK666). Enlarged F-actin and paxillin channels are shown below each 3D composite image.

(B) Average configuration (phalloidin brightness, density heatmaps) of N circular-shaped carcinoma cells.

(C) Morphometric analysis of alignment of (1) the cell (analysis of length and width) and (2) subcellular structures in lamellipodia (F-actin fiber alignment angle relative to nanolines).

(D) Focal adhesion area distribution. Plot widths represent frequency of a FA area. Data in bi-axial cell spreading plots are mean ± SD; **$p < 0.001$ (unpaired t tests)

MT network. Therefore, we suggest mechanically driven actomyosin-MT compaction as a well-suited mechanism for cell linearization on compliant nanolines.

To further explore mechanical and structural cytoskeleton dynamics during CG, we examined and perturbed MTs and contractility in cells on 1D microlines, which can mimic key

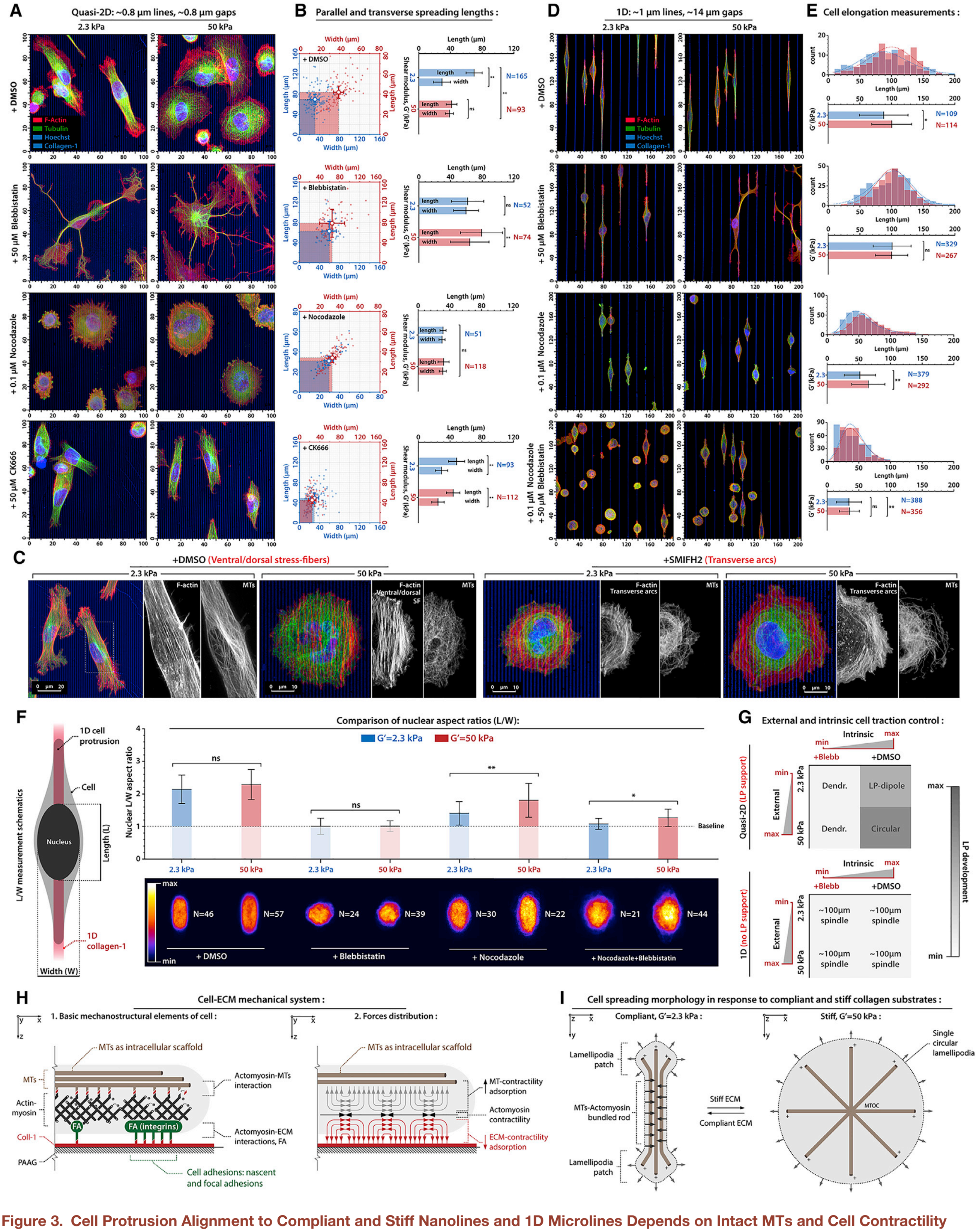

Figure 3. Cell Protrusion Alignment to Compliant and Stiff Nanolines and 1D Microlines Depends on Intact MTs and Cell Contractility

(A) Cell recognition and alignment to compliant and stiff nanolines in control conditions (+DMSO) and during actomyosin traction suppression (+Blebb), MTs disruption (+Nocodazole), and Arp2/3 suppression (+CK666). See Figure S4 for individual channels.

(legend continued on next page)

aspects of single 3D ECM fibers (Doyle et al., 2009). Sparse microlines do not allow multidirectional lamellipodia protrusions and thus allow us to explore MT-actomyosin behavior that is constrained along the CG cues. To establish a metric of effective actomyosin tension along cell axis, we measured nuclei deformation, where nucleus lateral compression results from cell tension alignment (Versaevel et al., 2012). Analysis of 1D protrusion and nuclei deformation in cells across both stiffnesses and contractility states (2.3 versus 50 kPa; ±Blebb) shows that protrusion activity is not dependent of effective traction (Figures 3D–3G). Alternatively, following MTs disruption (+Nocodazole) or MTs stabilization (+Taxol), or simultaneous disruption of contractility and MTs (Figures 3D–3F and S3A), 1D cell protrusion along lamellipodia-restricted microlines is significantly reduced, suggesting both MT-actomyosin compensation and cooperation. Thus, we conclude that there is a physical MT-actomyosin link where actomyosin-generated forces are mechanically adsorbed by both an MT intracellular scaffold and ECM (Figure 3H), resulting in the LP-dipole phenotype on compliant CG cues and a circular phenotype on stiff, flat CG cues (Figure 3I).

MT Scaffolds Regulate Steric Interactions with Nanotopographic CG Cues

To further define the role of MTs in CG-directed protrusions, we perturbed MT structure in cells responding to nanotextured substrates that induce robust cell orientation without the lateral constraints imposed by 1D microlines. Extrapolating from our data on flat quasi-2D nanolines and 1D microlines, the MT mechanical scaffold hypothesis suggests that MT-actomyosin interactions may help guide and mechanically enforce cell steric conformity to nanotextured CG cues and directed protrusion.

In response to nanotexture, MDA-MB-231 cells align and elongate along CG cues, consistent with our previous findings (Ray et al., 2017), with MT conformity to the nanogrooves (Figure 4A), a finding we confirmed in a distinct pancreatic ductal adenocarcinoma cell line (Figure S3F). Remarkably, we identified both "on-ridges" F-actin veils spanning atop multiple nanoridges as transverse arcs (TAs) with TA-localized MTs and "in-grooves" MT-scaffolded SF-rich projections (Figures 4A, 4B, and S5), consistent with early MT dependence for cell alignment conformation to large cell-sized titanium micrograting textures (Oakley and Brunette, 1995). Furthermore, 3D analysis of the cell-nanotexture interface demonstrates that highly aligned in-groove MTs emerge from the above on-ridge plane where the MT network is not as robustly oriented (Figure 4B). Thus, these collective observations suggest a regulatory role for in-groove protrusions, whereas the veil architectures suggest potential for a less sterically constrained CG cues sensing mode. Thus, we hypothesized that a dynamic MT network is being sterically trapped inside the nanogrooves, structurally regulating cell dentations and protrusion stability to enhance cell orientation and elongation along CG cues. Indeed, nocodazole effectively diminishes in-groove MTs, resulting in smooth-edged elliptic cells with decreased aspect ratios (length [L]/width [W], ~3 versus ~1.5) that are dynamically steady (Figures 4C and 4D; Video S4), unlike irregular-shaped MT-positive cells (Video S5). In addition, MT stabilization results in an even greater disruption to the CG response (Figure S3B). Therefore, we conclude that loss of dynamic in-groove MTs results in a more isotropic mode of protrusion, where cells are not as robustly directed by the nanotexture CG cues.

Competition between Arp2/3- and Formins-Dependent Actin Architectures Regulate MT-Dependent Protrusions that Promote the CG Response

Analysis of the actin and MT cytoskeletons in cells responding to nanotextured CG cues demonstrates that MTs are sterically trapped in nanogrooves with in-groove actin that extends into the on-ridge regions (Figures 4A, 4E, S4C, and S4D). Thus, our collective data (Figures 2, 3, and 4) suggest a model for competition between in-groove dentations that are sterically guided into unidirectional cytoskeletal protrusion dynamics and the layer atop of the nanotexture that is not sterically constrained and therefore can feature more isotropic MT and actomyosin spreading dynamics (Figure 4F). Indeed, further analysis of the in-groove and on-ridge layers shows FAs located both on ridges and in grooves, but distinct actin and MTs architectures at each level (Figures 4G–4I). Aligned F-actin and MTs are clearly colocalized in-grooves, while on-ridge actin is organized as TAs that bound a more isotropic MT network (Figures 4G–4K). Thus, we suggest a physical mechanism that governs cytoskeleton alignment in nanogrooves, where the nanotextured CG cues sterically trap and laterally constrain MT+actin-rich protrusions while cell thinning in protrusion regions constrains them in the Z axis.

While our data provide a physical mechanism that governs directionality of MT-dependent in-groove protrusions, the question of what processes may actively guide the MTs inside the

(B) Corresponding lengths and widths distributions for the conditions outlined in (A).

(C) Cell architectures on compliant and stiff nanolines in control (left, +DMSO) and in the presence of Formins inhibitor (right, +SMIFH2). Note the transition from dorsal and/or ventral stress fibers aligned to collagen nanolines to circular transverse arcs under Formins inhibition.

(D) The cellular response to 1D collagen microlines under control conditions (+DMSO), contractility suppression (+Blebb), MTs disruption (+Nocodazole), and combined nocodazole-plus-blebbistatin treatment.

(E) Corresponding 1D cell lengths distributions for the conditions outlined in (D).

(F) Density heatmaps of averaged nuclei L/W aspect ratios on 1D CG cues.

(G) Summary of the cell responses to either intrinsic or external modulation of effective traction.

(H) Overview of the MT-actomyosin-ECM mechanical system on polyacrylamide gels (PAAGs). (Left) Actomyosin-cytoskeleton interacts with the ECM via FAs and with MTs via steric and molecular adaptor-mediated interactions (i.e., actomyosin-MT entanglement). (Right) Actomyosin-generated forces are mechanically adsorbed by both the MT intracellular scaffold and ECM.

(I) MT-actomyosin interactions lead to MT bundling and consequent cell linearization (i.e LP-dipole) on soft collagen. Stiff ECM induces, predominantly, adsorption of actomyosin forces by the ECM via FA complexes and consequently multidirectional protrusion (i.e., circular morphology) with dispersed MTs. Data are mean ± SD; ns, no significant difference; *p < 0.05, **p < 0.001 (ANOVA with Tukey post hoc analysis).

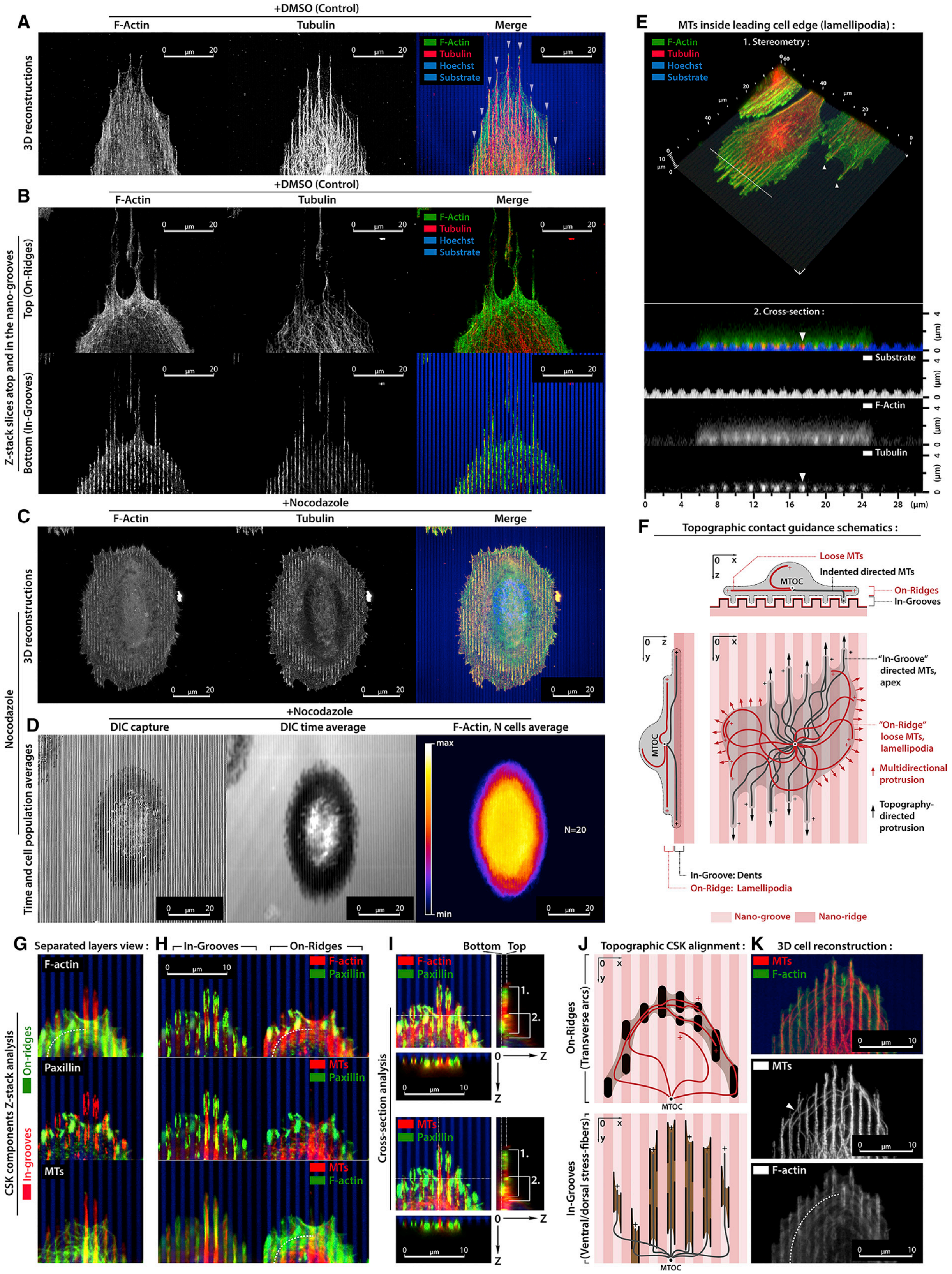

(legend on next page)

nanogrooves along the Z axis warrants further investigation. Thus, we sought to identify molecular regulators of the distinct cytoskeleton structures (i.e., aligned in-groove SFs versus on-ridge TAs) that may regulate competition between highly directed cell protrusions along CG cues and less directional lamellipodial protrusions. Suppression of intrinsic cell traction (+Blebb) decreases multidirectional lamellipodia and induces cell elongation and long, thin protrusions (Figures 5A and 5B), reminiscent of blebbistatin-induced "dendrites" observed on quasi-2D nanolines (Figure 3A). The increase of cell "width" results from long protrusions that are not well aligned to CG cues, consistent with decreased directed migration along nanotextured CG cues following blebbistatin treatment (Ray et al., 2017). Nevertheless, protrusions and blebbistatin-induced dendrites largely retain small MT-rich protrusions that sterically align to the nanogrooves (Figures S3G, S4C, and S4D). To confirm this finding, we developed compliant and stiff nanotextured polyacrylamide (PAA)-based substrates to facilitate overall lower effective traction forces across FAs via the softer substrates (Figure 5C). Notably, MT-rich in-groove protrusions are present for both stiffnesses. However, analysis of cell protrusions indeed display modulation of on-ridge lamellipodial spreading, with cells on stiff substrates developing greater on-ridge lamellipodia both along and transverse to the nanogrooves, while cells on the compliant nanotextures demonstrate less on-ridge lamellipodia spreading, particularly along the cell body (Figures 5C and 5D), which is consistent with our findings on flat nanolines, and in 1D protrusion where scaffolding MTs render protrusions insensitive to external CG cue rigidity. Thus, the decrease in on-ridge spreading and maintenance of MT-positive in-groove protrusions demonstrates that decreasing cell contractility disrupts multidirectional on-ridge lamellipodia dynamics but is not required for MT-positive in-groove protrusions. Therefore, we hypothesized that signaling pathways that robustly shift the balance between dorsal and/or ventral SFs and on-ridge TAs drive a signaling-structure-function relationship where actin cytoskeleton architecture governs MT dynamics.

Altering actin cytoskeleton structure by manipulating Arp2/3 and Formins signaling does indeed regulate MT dynamics that promote the sensing of CG cues. Targeting Arp2/3 decreases TAs in favor of increasing aligned in-groove SFs and MT (Figure S5). This change in lamellipodia dynamics results in robust cell alignment along CG cues for both contractile and low traction cells (i.e., ±Blebb) and loss of blebbistatin-induced dendritic protrusions following Arp2/3 inhibition (Figures 5A, 5B, and S4). Indeed, Arp2/3 suppression of on-ridge lamellipodia dynamics leads to cell linearization similar to findings on quasi-2D nanolines (Figures 2 and 3), whereas nocodazole-treatment results in no principal shift in cell architecture (i.e., cells remain elliptic) between contractile and blebbistatin-treated cells (Figures 5A and 5B). Similar to the cell phenotype after MT disruption, inhibition of Formins, which converts the actin cytoskeleton to the TAs architecture (Figure S5B), profoundly decreases the CG response (Figures 5A, 5B, and S4C). Notably, this shift in actin architecture largely results in loss of in-groove MT protrusions (Figures S4D and S5). In fact, quantification of in-groove versus on-ridge protrusions across +SMIFH2;+DMSO;+CK666 treatments (Figure 5E) clearly demonstrates a shift from on-ridge to in-groove behavior that results from altering actin architecture (Figure S5B), which regulates in-groove MT dynamics (Figure S5C). Thus, we suggest that aligned in-groove MT scaffolds are a key regulator of the CG sensing response and that MT localization to nanogrooves is regulated by the Arp2/3- or Formin-dependent balance between SF and TA architectures associated with more directed versus multidirectional protrusions.

DISCUSSION

Here, we determined that MTs are structurally and mechanically involved in regulation of CG sensing on both flat (quasi-2D and 1D) and sterically active (nanotopography) CG cues. On 2D nanolines, MTs influence cell shape through biomechanical competition for actomyosin contractile energy with the integrin-ECM mechanical system. We show that this competition can be described in terms of a balance between multidirectional lamellipodia spreading, promoted by stiff substrates, and actomyosin compaction (i.e., collapse) of MT-scaffolded bundles into rods (i.e., linearization) on compliant CG substrates, which induce lower traction forces and thus understimulated actomyosin traction to allow compaction. Likewise, non-pharmacological suppression of multidirectional lamellipodia via CG architectures (1D microlines) or pharmacologic lamellipodia suppression (+CK666) leads to structural convergence of actomyosin and MTs, enhancing their mechanical and architectural cooperation. Likewise, MTs also serve as active intracellular scaffolds during steric interactions with nanotopographic features (i.e., ECM

Figure 4. MTs Regulate Sterically Trapped Nanogroove Protrusions to Promote the CG Response

(A) Aligned in-groove MTs in multiple apices (arrowheads) at the cell front.

(B) On-ridge and in-groove cell layers with aligned F-actin and MTs confined in nanogrooves and on-ridge F-actin transverse arcs constraining less organized MTs.

(C) Disruption of MTs (+Nocodazole) suppresses in-groove apical protrusions, resulting in elliptic cells with decreased CG response.

(D) DIC capture, time average (180 min), and heatmap analysis of live-cell shape during nocodazole treatment.

(E) 3D stereometric view and vertical cross-section (dashed line) of the cell leading edge interacting with nanotexture CG cues. Note that MTs and actin indented into the nanogrooves (arrowhead).

(F) Schematic of hypothesized competitive dynamics between in-groove SF-MT-regulated guidance and on-ridge lamellipodia spreading.

(G) F-actin, paxillin, and MTs structures in the on-ridge (green) and in-groove (red) layers. Note that the transverse arcs within the on-ridge layer (dashed line).

(H) F-actin+paxillin, MT+paxillin, and MT+F-actin signals within in-groove and on-ridge layers (dashed line, transverse arcs).

(I) 3D reconstructions and X0Z and Y0Z cross-sections (along dashed lines) reveals progressive cell thinning and "sinking" from the on-ridge-plus-in-groove structures (1 and 2) to the in-groove (1) protrusions at the cell periphery. Note the dorsal and/or ventral SFs (1) located inside the nanogrooves.

(J) Schematic view of on-ridge transverse arcs and in-groove dorsal and/or ventral SFs that trap MTs in the corresponding layers.

(K) 3D cell reconstruction visualizing in-groove and on-ridge MTs. Note the on-ridge MTs (white arrowhead) can interact with transverse arcs (dashed line).

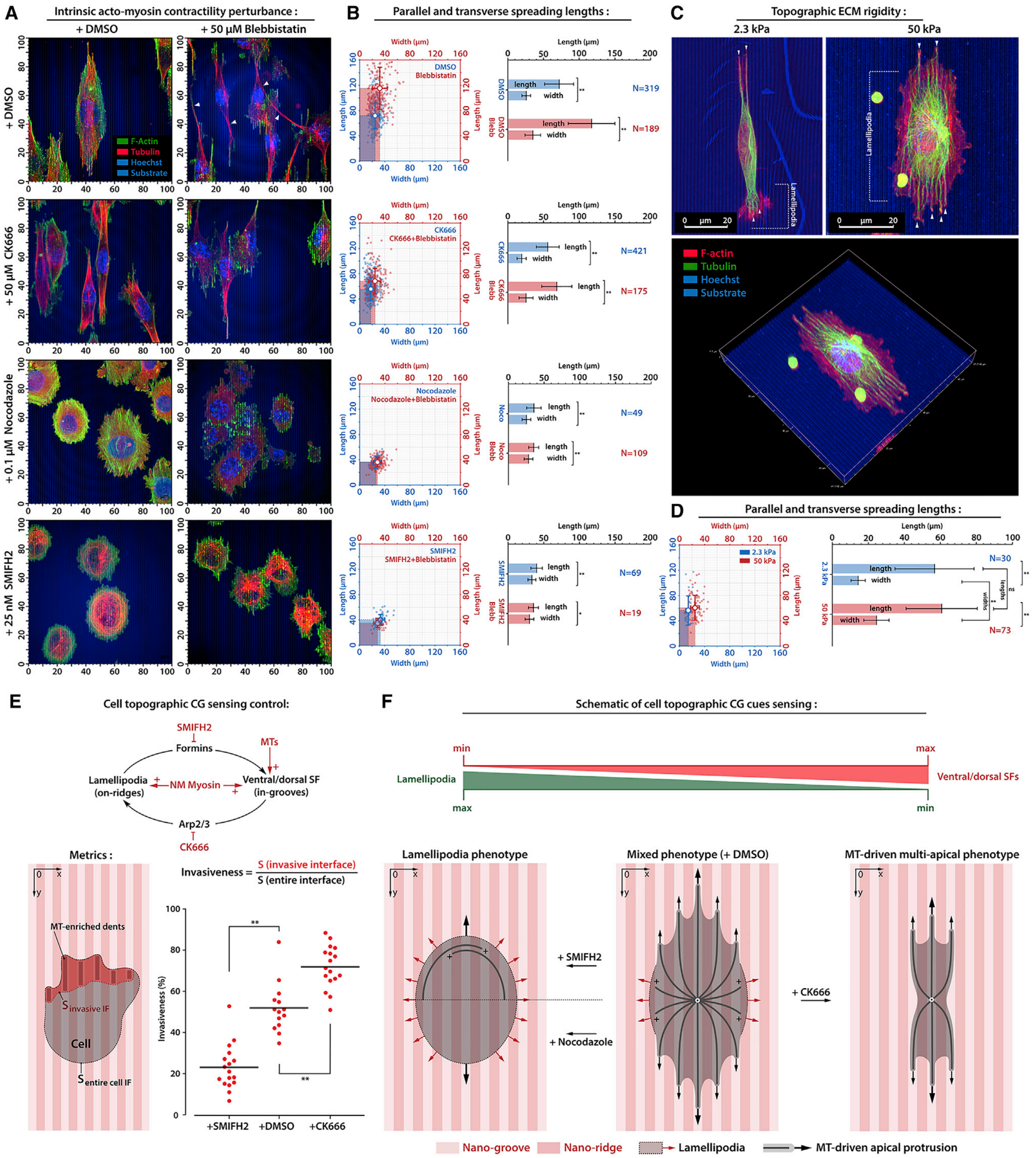

Figure 5. Arp2/3- and Formins-Dependent Actin Architectures Regulate MT-Dependent Protrusions that Promote the CG Response

(A) Contractile (+DMSO) and actomyosin contraction inhibited (+Blebb) cells under control conditions (top row), Arp2/3 inhibition (+CK666), MTs disruption (+Nocodazole) or Formins inhibition (+SMIFH2). See Figure S4 for individual channels and cross-sections of protrusions into nanogrooves.

(B) Population views and mean values for lengths along and widths across nanolines for the conditions outlined in (A).

(C) (Top) 3D reconstructions of cell protrusion along compliant (2.3kPa) and stiff (50kPa) collagen-coated PAA nanogroove substrates. (Bottom) Stereometric view.

(D) Lengths and widths for conditions in (C). Note that cells on both stiffnesses produce MT-rich in-groove protrusions, while on stiff substrates on-ridge lamellipodial protrusions are more robust.

(legend continued on next page)

nanolandscapes can laterally trap MTs and reinforce them inside nanogrooves, consistent with laterally reinforced MTs bearing load [Brangwynne et al., 2006]) where the balance between Arp2/3-dependent on-ridge TAs and Formins-dependent in-groove dorsal and/or ventral SFs that appear to actively guide MTs inside the nanotextures. Indeed, during competitive on-ridge lamellipodia spreading, F-actin translocation to the on-ridge layer consequently disables MT-nanogrooves steric interactions, as shown with SMIFH2-induced F-actin transition toward an on-ridge TA-dominated architecture. Conversely, suppression of on-ridge multidirectional lamellipodia dynamics enhances in-groove F-actin SF-like structures that promote sterically guided MTs and greater cell-CG alignment. Thus, we identified a regulatory balance between TA and dorsal and/or ventral SF actin architectures (Hotulainen and Lappalainen, 2006; Oakes et al., 2012), as their balance determines MT-dependent sensing of topographic CG cues. As such, the collective data presented here add considerable insight into the mechanisms governing sensing of aligned collagen matrices, which are known to direct breast carcinoma cell invasion (Conklin et al., 2011; Patsialou et al., 2013; Provenzano et al., 2006), and suggest that, in addition to their well-established roles in targeting proliferation, MT targeting agents likely impact carcinoma cell sensing of CG cues. Furthermore, these data suggest that targeting distinct Formins may provide a rational strategy for disrupting metastatic behavior.

STAR★METHODS

Detailed methods are provided in the online version of this paper and include the following:

- KEY RESOURCES TABLE
- CONTACT FOR REAGENT AND RESOURCE SHARING
- EXPERIMENTAL MODEL AND SUBJECT DETAILS
  - Experimental models
- METHOD DETAILS
  - Principles of high precision patterning
  - Stamp-casting for nano/micro-matrices
  - Coating and labeling nano/micro-stamps
  - PAA elastic gels premixes
  - Micro- and nano-contact printing
  - Preparation of elastic nanotextured CG cues
  - Cell Contact Guidance Assays
  - Imaging
  - Cell traction forces analysis
  - Statistical Analysis

SUPPLEMENTAL INFORMATION

Supplemental Information includes five figures and five videos and can be found with this article online at https://doi.org/10.1016/j.celrep.2018.09.030.

ACKNOWLEDGMENTS

P.P.P. and this work were supported by a Research Scholar Grant (RSG-14-171-01-CSM) from the American Cancer Society and by the NIH (U54CA210190 University of Minnesota Physical Sciences in Oncology Center Project 2 to P.P.P., R01CA181385 to P.P.P., and R01CA181385S1 to E.D.T. and P.P.P.), UMN College of Science and Engineering (P.P.P.), Masonic Cancer Center (P.P.P.), and grants from the UMN Institute for Engineering in Medicine (P.P.P.) and the Randy Shaver Research and Community Fund (P.P.P.). The content of this work is solely the responsibility of the authors and does not necessarily represent the official views of the NIH or other funding agencies. The University Imaging Center (http://uic.umn.edu) at the University of Minnesota facilitates use of the Nikon A1Rsi Confocal microscope and its integral 2D and 3D image analysis software (NIS-Elements Confocal, NIS-Elements Confocal Advanced Research). We thank University Imaging Center staff, and in particular Dr. Guillermo Marques, for helpful assistance. Portions of this work were conducted in the Minnesota Nano Center, which is supported by the National Science Foundation through the National Nano Coordinated Infrastructure Network (NNCI) under Award ECCS-1542202. We thank Zaw Win for helpful assistance with imaging. We thank the members of the Provenzano laboratory for insightful comments regarding this work.

AUTHOR CONTRIBUTIONS

E.D.T. participated in the design, execution, and analysis of all micropatterning or nanopatterning and experiments. E.D.T. developed micropatterning methods and all platforms employed in this study. V.P. performed experiments and analysis. A.Z. participated in experimental design, data analysis, and data interpretation. P.P.P. participated in experimental design, data analysis, data interpretation, and secured funding. E.D.T. and P.P.P. wrote the manuscript. All authors read and contributed comments to the final manuscript. E.D.T. and P.P.P. oversaw all aspects of the study.

DECLARATION OF INTERESTS

The authors declare no competing interests.

Received: June 14, 2018
Revised: August 20, 2018
Accepted: September 7, 2018
Published: October 9, 2018

REFERENCES

Albuschies, J., and Vogel, V. (2013). The role of filopodia in the recognition of nanotopographies. Sci. Rep. *3*, 1658.

Bouchet, B.P., and Akhmanova, A. (2017). Microtubules in 3D cell motility. J. Cell Sci. *130*, 39–50.

Brangwynne, C.P., MacKintosh, F.C., Kumar, S., Geisse, N.A., Talbot, J., Mahadevan, L., Parker, K.K., Ingber, D.E., and Weitz, D.A. (2006). Microtubules can bear enhanced compressive loads in living cells because of lateral reinforcement. J. Cell Biol. *173*, 733–741.

Burnette, D.T., Ji, L., Schaefer, A.W., Medeiros, N.A., Danuser, G., and Forscher, P. (2008). Myosin II activity facilitates microtubule bundling in the neuronal growth cone neck. Dev. Cell *15*, 163–169.

Carey, S.P., Starchenko, A., McGregor, A.L., and Reinhart-King, C.A. (2013). Leading malignant cells initiate collective epithelial cell invasion in a

(E) Schematic and plot of metrics capturing in-groove protrusive invasiveness that decreases from Formins inhibition and increases from Arp2/3-inhibition, where Formins and Arp2/3 regulate the transition between ventral and/or dorsal SFs and transverse arcs to regulate in-groove MTs that promote directed protrusion and the response to CG (see Figure S5).

(F) Schematic of competitive dynamics between on-ridge lamellipodial and in-groove MT-driven apical nanogroove-guided protrusions. Data are mean ± SD; ns, no significant difference; *p < 0.05, **p < 0.001.

three-dimensional heterotypic tumor spheroid model. Clin. Exp. Metastasis *30*, 615–630.

Carey, S.P., Rahman, A., Kraning-Rush, C.M., Romero, B., Somasegar, S., Torre, O.M., Williams, R.M., and Reinhart-King, C.A. (2015). Comparative mechanisms of cancer cell migration through 3D matrix and physiological microtracks. Am. J. Physiol. Cell Physiol. *308*, C436–C447.

Chang, S.S., Guo, W.-H., Kim, Y., and Wang, Y.-L. (2013). Guidance of cell migration by substrate dimension. Biophys. J. *104*, 313–321.

Conklin, M.W., Eickhoff, J.C., Riching, K.M., Pehlke, C.A., Eliceiri, K.W., Provenzano, P.P., Friedl, A., and Keely, P.J. (2011). Aligned collagen is a prognostic signature for survival in human breast carcinoma. Am. J. Pathol. *178*, 1221–1232.

Daley, W.P., and Yamada, K.M. (2013). ECM-modulated cellular dynamics as a driving force for tissue morphogenesis. Curr. Opin. Genet. Dev. *23*, 408–414.

Doyle, A.D., Wang, F.W., Matsumoto, K., and Yamada, K.M. (2009). One-dimensional topography underlies three-dimensional fibrillar cell migration. J. Cell Biol. *184*, 481–490.

Doyle, A.D., Kutys, M.L., Conti, M.A., Matsumoto, K., Adelstein, R.S., and Yamada, K.M. (2012). Micro-environmental control of cell migration–myosin IIA is required for efficient migration in fibrillar environments through control of cell adhesion dynamics. J. Cell Sci. *125*, 2244–2256.

Dugina, V., Alieva, I., Khromova, N., Kireev, I., Gunning, P.W., and Kopnin, P. (2016). Interaction of microtubules with the actin cytoskeleton via cross-talk of EB1-containing +TIPs and γ-actin in epithelial cells. Oncotarget *7*, 72699–72715.

Fischer, R.S., Myers, K.A., Gardel, M.L., and Waterman, C.M. (2012). Stiffness-controlled three-dimensional extracellular matrices for high-resolution imaging of cell behavior. Nat. Protoc. *7*, 2056–2066.

Friedl, P., and Bröcker, E.B. (2000). T cell migration in three-dimensional extracellular matrix: guidance by polarity and sensations. Dev. Immunol. *7*, 249–266.

Gardel, M.L., Kasza, K.E., Brangwynne, C.P., Liu, J., and Weitz, D.A. (2008). Chapter 19: mechanical response of cytoskeletal networks. Methods Cell Biol. *89*, 487–519.

Guetta-Terrier, C., Monzo, P., Zhu, J., Long, H., Venkatraman, L., Zhou, Y., Wang, P., Chew, S.Y., Mogilner, A., Ladoux, B., and Gauthier, N.C. (2015). Protrusive waves guide 3D cell migration along nanofibers. J. Cell Biol. *211*, 683–701.

Hotulainen, P., and Lappalainen, P. (2006). Stress fibers are generated by two distinct actin assembly mechanisms in motile cells. J. Cell Biol. *173*, 383–394.

Jacquemet, G., Hamidi, H., and Ivaska, J. (2015). Filopodia in cell adhesion, 3D migration and cancer cell invasion. Curr. Opin. Cell Biol. *36*, 23–31.

Lee, M.-H., Wu, P.-H., Gilkes, D., Aifuwa, I., and Wirtz, D. (2015). Normal mammary epithelial cells promote carcinoma basement membrane invasion by inducing microtubule-rich protrusions. Oncotarget *6*, 32634–32645.

Lee, K., Kim, E.H., Oh, N., Tuan, N.A., Bae, N.H., Lee, S.J., Lee, K.G., Eom, C.-Y., Yim, E.K., and Park, S. (2016). Contribution of actin filaments and microtubules to cell elongation and alignment depends on the grating depth of microgratings. J. Nanobiotechnology *14*, 35.

Nuhn, J.A.M., Perez, A.M., and Schneider, I.C. (2018). Contact guidance diversity in rotationally aligned collagen matrices. Acta Biomater. *66*, 248–257.

Oakes, P.W., Beckham, Y., Stricker, J., and Gardel, M.L. (2012). Tension is required but not sufficient for focal adhesion maturation without a stress fiber template. J. Cell Biol. *196*, 363–374.

Oakley, C., and Brunette, D.M. (1995). Topographic compensation: guidance and directed locomotion of fibroblasts on grooved micromachined substrata in the absence of microtubules. Cell Motil. Cytoskeleton *31*, 45–58.

Odom, T.W., Love, J.C., Wolfe, D.B., Paul, K.E., and Whitesides, G.M. (2002). Improved pattern transfer in soft lithography using composite stamps. Langmuir *18*, 5314–5320.

Patsialou, A., Bravo-Cordero, J.J., Wang, Y., Entenberg, D., Liu, H., Clarke, M., and Condeelis, J.S. (2013). Intravital multiphoton imaging reveals multicellular streaming as a crucial component of in vivo cell migration in human breast tumors. Intravital *2*, e25294.

Picone, R., Ren, X., Ivanovitch, K.D., Clarke, J.D.W., McKendry, R.A., and Baum, B. (2010). A polarised population of dynamic microtubules mediates homeostatic length control in animal cells. PLoS Biol. *8*, e1000542.

Plotnikov, S.V., Sabass, B., Schwarz, U.S., and Waterman, C.M. (2014). High-resolution traction force microscopy. Methods Cell Biol. *123*, 367–394.

Polio, S.R., and Smith, M.L. (2014). Patterned hydrogels for simplified measurement of cell traction forces. Methods Cell Biol. *121*, 17–31.

Provenzano, P.P., Eliceiri, K.W., Campbell, J.M., Inman, D.R., White, J.G., and Keely, P.J. (2006). Collagen reorganization at the tumor-stromal interface facilitates local invasion. BMC Med. *4*, 38.

Provenzano, P.P., Inman, D.R., Eliceiri, K.W., Trier, S.M., and Keely, P.J. (2008). Contact guidance mediated three-dimensional cell migration is regulated by Rho/ROCK-dependent matrix reorganization. Biophys. J. *95*, 5374–5384.

Ramirez-San Juan, G.R., Oakes, P.W., and Gardel, M.L. (2017). Contact guidance requires spatial control of leading-edge protrusion. Mol. Biol. Cell *28*, 1043–1053.

Ray, A., Lee, O., Win, Z., Edwards, R.M., Alford, P.W., Kim, D.-H., and Provenzano, P.P. (2017). Anisotropic forces from spatially constrained focal adhesions mediate contact guidance directed cell migration. Nat. Commun. *8*, 14923.

Rhee, S., Jiang, H., Ho, C.-H., and Grinnell, F. (2007). Microtubule function in fibroblast spreading is modulated according to the tension state of cell-matrix interactions. Proc. Natl. Acad. Sci. USA *104*, 5425–5430.

Robison, P., Caporizzo, M.A., Ahmadzadeh, H., Bogush, A.I., Chen, C.Y., Margulies, K.B., Shenoy, V.B., and Prosser, B.L. (2016). Detyrosinated microtubules buckle and bear load in contracting cardiomyocytes. Science *352*, aaf0659.

Romsey, N.R., Hou, Y., Rodriguez, L.L., and Schneider, I.C. (2014). The number of lines a cell contacts and cell contractility drive the efficiency of contact guidance. Cell. Mol. Bioeng. *7*, 122–135.

Schmid, H., and Michel, B. (2000). Siloxane polymers for high-resolution, high-accuracy soft lithography. Macromolecules *33*, 3042–3049.

Tabdanov, E., Gondarenko, S., Kumari, S., Liapis, A., Dustin, M.L., Sheetz, M.P., Kam, L.C., and Iskratsch, T. (2015). Micropatterning of TCR and LFA-1 ligands reveals complementary effects on cytoskeleton mechanics in T cells. Integr. Biol. *7*, 1272–1284.

Tang, X., Ali, M.Y., and Saif, M.T.A. (2012). A novel technique for micro-patterning proteins and cells on polyacrylamide gels. Soft Matter *8*, 7197–7206.

Teixeira, A.I., Abrams, G.A., Bertics, P.J., Murphy, C.J., and Nealey, P.F. (2003). Epithelial contact guidance on well-defined micro- and nanostructured substrates. J. Cell Sci. *116*, 1881–1892.

Tseng, Q., Duchemin-Pelletier, E., Deshiere, A., Balland, M., Guillou, H., Filhol, O., and Théry, M. (2012). Spatial organization of the extracellular matrix regulates cell-cell junction positioning. Proc. Natl. Acad. Sci. USA *109*, 1506–1511.

Versaevel, M., Grevesse, T., and Gabriele, S. (2012). Spatial coordination between cell and nuclear shape within micropatterned endothelial cells. Nat. Commun. *3*, 671.

STAR★METHODS

KEY RESOURCES TABLE

REAGENT or RESOURCE	SOURCE	IDENTIFIER
Antibodies		
Anti-Collagen I polyclonal antibody, Rabbit	AbCam	Cat# ab34710; RRID: AB_731684
Anti-Tubulin monoclonal antibody [YL1/2], Rat	AbCam	Cat# ab6160; RRID: AB_305328
Anti-Tubulin monoclonal antibody labeled with Alexa Fluor 488 [YL1/2], Rat	AbCam	Cat# ab197737
Purified Anti-Paxillin Clone 349/Paxillin (RUO), Mouse	BD BioSciences	Cat# 610052; RRID:AB_397464
Monoclonal Anti-β-Actin antibody, Mouse	Sigma-Aldrich	Cat# A5441; RRID: AB_476744
Alexa Fluor 568 goat anti-rat IgG (H+L)	Thermo Fisher	Cat# 11077; RRID: AB_2534121
Alexa Fluor 488 donkey anti-rat IgG (H+L)	Thermo Fisher	Cat# a21208; RRID: AB_141709
Alexa Fluor 568 goat anti-mouse IgG (H+L)	Thermo Fisher	Cat# A11004; RRID: AB_2534072
Alexa Fluor 488 goat anti-mouse IgG (H+L)	Thermo Fisher	Cat# 21131; RRID: AB_2535771
Chemicals, Peptides, and Recombinant Proteins		
HMS-31, (25%-35% Methylhydrosiloxane)- Dimethylsiloxane Copolymer, Trimethylsiloxane Terminated	Gelest	Cat# HMS-301; CAS#68037-59-2
VDT-731, (7.0%-8.0% Vinylmethylsiloxane) - Dimethylsiloxane Copolymer, Trimethylsiloxy Terminated	Gelest	Cat# VDT-731; CAS#67762-94-1
2,4,6,8-Tetramethyl-2,4,6,8-tetravinylcyclotetrasiloxane	Sigma-Aldrich	Cat# 396281; CAS#2554-06-5
Platinum(0)-2,4,6,8-tetramethyl-2,4,6,8-tetravinylcyclotetrasiloxane complex solution	Sigma-Aldrich	Cat# 479543; CAS#68585-32-0
SYLGARD 184 Silicone Elastomer Kit, 0.5 KG KIT	Dow Corning, Sigma-Aldrich	Cat# 4019862; CAS#68988-89-6
40% Acrylamide Solution, Electrophoresis purity reagent, 500mL	BioRad	Cat# 161-0140
2% Bis Solution, 500mL	BioRad	Cat# 161-0142
Streptavidin Acrylamide, 1mg	Life Technologies	Cat# S21379
TEMED	Thermo Scientific	Cat# 17919; CAS#110-18-9
Ammonium Persulfate, BioUltra, for molecular biology	Fluka Analytical	Cat# 09913-100G; CAS#7727-54-0
3-(Trimethoxysilyl)propyl methacrylate	Sigma-Aldrich	Cat# 6514; CAS#2530-85-0
Ethyl Alcohol 200 Proof, Absolute, Anhydrous ACS/USP Grade	Pharmco-Aaper	Cat# 111000200; CAS#64-17-5
Collagen Type I, Rat Tail High Concentration, 100 MG, 8.95 mg/mL	Corning	Cat# 354249
CK666, Arp2/3 inhibitor, 2-Fluoro-*N*-[2-(2-methyl-1*H*-indol-3-yl)ethyl]benzamide	Tocris	Cat# 3950; CAS#442633-00-3
(-)-Blebbistatin, 1-Phenyl-1,2,3,4-tetrahydro-4-hydroxypyrrolo[2.3-b]-7-methylquinolin-4-one	Sigma-Aldrich	Cat# 203391; CAS#856925-71-8
Nocodazole, MT Inhibitor	AbCam	Cat# ab120630; CAS#31430-18-9
SMIFH2 Formin FH2 Domain Inhibitor	AbCam	Cat# ab218296; CAS#340316-62-3
Paclitaxel (Taxol)	Sigma-Aldrich	Cat# T7402; CAS#33069-62-4
Methanol, HPLC pure, >99.9%	Sigma-Aldrich	Cat# 34860-4L-R; CAS#67-56-1
Triton X-100	Sigma-Aldrich (Roche)	Cat# 11332481001 CAS#9002-93-1
Paraformaldehyde, reagent grade, crystalline	Sigma-Aldrich	Cat# P6148-500G; CAS#30525-89-4
Hoechst 33342, Fluorescent Dye for labeling DNA	Tocris	Cat# 5117; CAS#23491-52-3
Bovine Serum Albumin (BSA), fatty acid-free powder	Fisher Bioreagents	Cat# BP9704-100; CAS#9048-46-8
PBS pH7.4 (1X), Phosphate Buffer Saline	GIBCO	Cat# 10010-023
DMEM, 1X (Dulbecco's Modification of Eagle's Medium) with 4.5 g/L glucose, L-glutamine & sodium pyruvate	Corning Cellgro	Cat# 10-013-CV
0.25% Trypsin, 2.21 mM EDTA, 1X [-] sodium bicarbonate	Corning	Cat# 25-053-CI
Penicillin Streptomycin Solution, 100X	Corning	Cat# 30-002-CI
Fetal Bovine Serum	HyClone	Cat# SH30910.03

(*Continued on next page*)

Continued		
REAGENT or RESOURCE	SOURCE	IDENTIFIER
Acetic Acid, Glacial	Fisher Chemical	Cat# BP2401-500; CAS#64-19-7
DMSO (Dimethyl sulfoxide)	Sigma-Aldrich	Cat# 472301-100ML; CAS#67-68-5
Silanization solution I	Sigma-Aldrich	Cat# 85126; CAS#75-78-5
0.2 μm Red Fluorescent Beads	Polysciences	Cat# BLI832-1
Sodium Dodecyl Sulfate (SDS)	Fisher Bioreagents	Cat# BP166-100;CAS#151-21-3
0.22 μm Millex GP	Millipore-Sigma	Cat# SLGP033NS
Cover Glasses, Circles, 15 mm, Thickness 0.13-0.17 mm	Carolina Biological Supply Company	Cat# 633031
Critical Commercial Assays		
Slide-A-Lyzer MINI Dialysis Device, 7K MWCO, 0.1 mL	Thermo Fisher	Cat# 69560
(+)-Biotin N-hydroxysuccinimide ester	Sigma-Aldrich	Cat# H1759; CAS#35013-72-0
Alexa Fluor 488 carboxic acid, succinimidyl ester	Molecular Probes	Cat# A20000
Alexa Fluor 568 carboxic acid, succinimidyl ester	Molecular Probes	Cat# A20003
Phalloidin-iFluor 647 Reagent - CytoPainter	AbCam	Cat# ab176759
Phalloidin-iFluor 488 Reagent - CytoPainter	AbCam	Cat# ab176753
Experimental Models: Cell Lines		
Human breast adenocarcinoma cell line MDA-MB-231 (ATCC HTB-26), Female	ATCC	Cat# HTB-26; RRID:CVCL_0062
Human Pancreatic Adenocarcinoma cell line MIA PaCa-2 (ATCC CRL-1420), Male	ATCC	Cat# CRL-1420; RRID:CVCL_0428
Software and Algorithms		
NIS-Elements Advanced Research 3.0	Nikon Instruments	RRID: SCR_014329
NIS-Elements Confocal software 3.0	Nikon Instruments	RRID: SCR_002776
FIJI (ImageJ), Version: 2.0.0-rc-54/1.51h	https://fiji.sc/#	RRID: SCR_002285
PIV (Particle Image Velocimetry)	https://sites.google.com/site/qingzongtseng/piv	N/A
KaleidaGraph 4.5.3	http://www.synergy.com/wordpress_650164087/	RRID: SCR_014980
GraphPad Prism 7b	https://www.graphpad.com/	RRID: SCR_002798
Adobe Photoshop CC, 20161012.r.53x 64	Adobe Systems	RRID: SCR_014199
Adobe Illustrator CC, 21.0.0.	Adobe Systems	RRID: SCR_010279

CONTACT FOR REAGENT AND RESOURCE SHARING

Further information and requests for resources and reagents should be directed to and will be fulfilled by the Lead Contact, Paolo Provenzano (pprovenz@umn.edu)

EXPERIMENTAL MODEL AND SUBJECT DETAILS

Experimental models

Human breast (MDA-MB-231: Female human breast adenocarcinoma cell line, ATCC® HTB-26) and pancreatic (MIA-Paca-2: Male Human Pancreatic Adenocarcinoma cell line ATCC® CRL-1420), were freshly obtained from the ATCC cell bank, where they were validated, at the start of these studies and were used within 10 passages from initial cultures, with no deviation in phenotype, while remaining free of Mycoplasma, for all experiments. Both lines were maintained in DMEM with 4.5 g/L D-glucose, L-glutamine,110 mg/L sodium pyruvate (Corning Cellgro®, USA) and 10% heat-inactivated FBS (HyClone®, USA) at 37C in 5% CO2. All cell work was approved by the University of Minnesota Institutional Biosafety Committee and followed institutional and NIH guidelines.

METHOD DETAILS

Principles of high precision patterning

Fabrication of elastic collagen nano- and micro-patterns is a challenging task due to the susceptibility of type-I collagen to undergo rapid gelation, and van-der-waals and capillary interactions between the nano-stamp and the printed surface that provoke a collapse

of the soft PDMS nano-stamps onto the glass surface. To address these issues and achieve high precision micro- and nano-patterns on elastic platforms we (i) substituted regular PDMS nano-stamps with composite stamps, veneered with a submillimeter-thick hard PDMS (hPDMS) for non-collapsing high-definition printing surfaces (Schmid and Michel, 2000; Tabdanov et al., 2015), and (ii) substituted collagen with α-collagen-1 rabbit pAb (AbCam, Cambridge, UK), conjugated with biotin and a fluorescent tag, to ensure cross-linking of the antibody to PAA gels and for fluorescence visibility, respectively. For hPDMS we mixed 3.4g of VDT-731 (Gelest, Inc.), 18μL of Pt catalyst (Platinum(0)-2,4,6,8-tetramethyl-2,4,6,8-tetravinylcyclotetrasiloxane complex solution) (Sigma-Aldrich) and one drop of cross-linking modulator (2,4,6,8-Tetramethyl-2,4,6,8-tetravinylcyclotetrasiloxane) (Sigma-Aldrich). Next, immediately before use, we added 1g of HMS-301 (Gelest, Inc.) and thoroughly mixed it for 30sec on vortex mixer (Odom et al., 2002).

Stamp-casting for nano/micro-matrices

In order to cast the nano-printing surface, we used commercially manufactured polyurethane nano-surfaces as the casting matrices (NanoSurface Biomedical, Seattle, WA). Clean textured nano-surface (NanoSurface Biomedical, Seattle, WA) disks were glued onto the glass platform with SuperGlue® (Loctite, USA), silanized with silanizing solution-I as per the commercial protocol (Sigma Aldrich), coated with ≤ 0.5mm hPDMS by gentle spreading with soft Parafilm-made spatula (Hach, USA), cured at 70°C for 30 minutes and subsequently cast with regular PDMS to the layer final thickness of 8mm (rPDMS; 1:5 curing agent/base ratio, Sylgard-184, Dow Corning). Cured (at 70°C for ~1 hour) composite nano-stamps were peeled, and cut into 5×5mm or 1×1cm pieces and used as the ready-to-use nano-stamps. For microprinting 1D microlines, the casting matrix for 1μm-wide and 15μm-pitched microline patterns was designed and commercially manufactured using customized UV photolithography (UMN NanoCenter, MN, USA).

Coating and labeling nano/micro-stamps

Anti-collagen-1 rabbit pAb (AbCam, Cambridge, UK) was prelabeled with a fluorescent tag and a biotin group to ensure both its fluorescent visibility in nanopatterns and cross-linking to the streptavidin-functionalized PAA gels, respectively. Briefly, 20μL of 1mg/mL antibody sample was incubated for 1 hour with 5μL of ((+)-biotin N-hydroxysuccinimide ester, Sigma-Aldrich; as per the commercial protocol) and 5μL of fluorescent tag kit (Alexa Fluor® succinimidyl esters, Invitrogen, Molecular Probes®; as per the commercial protocol). Labeled protein then was dialysed overnight in Slide-A-Lyzer MINI Dialysis Device, 7K MWCO (Thermo Fisher) overnight at 4°C in cold PBS, then stored at 4°C in the darkness. 10μL droplets of 0.1mg/mL labeled antibody solution were then placed atop of the 5×5mm or 1×1cm square micro- or nano-stamps. To ensure a proper coverage and effective stamp surface coating with labeled α-collagen-1 antibody, the antibody solution droplet was "sandwiched" between the stamp's printing surface and 15mm round glass coverslip (Carolina, USA), which had been baked in the furnace for 5-10 hours at 450°C.

PAA elastic gels premixes

We chose to control PAA mechanical rigidity via modulation of concentration for both 40% acrylamide (40% AA) base (BioRad) and its cross-linking molecular chain, 2% bis-AA (BioRad) as described elsewhere (Fischer et al., 2012; Plotnikov et al., 2014). Additionally, streptavidin-acrylamide (Thermo Fisher) was added to the final concentration of 0.133mg/mL to enable PAA gels cross-linking with biotinylated proteins of interest. Briefly, for preparation of 50μL of G' = 2.3 and 50kPa PAA gel premixes, respectively, the following components were mixed: 40% AA: 9.33 and 15μL; 2% bis-AA: 1.88 and 14.40μL; 2mg/mL streptavidin-AA: 3.33 and 3.33μL; 10X PBS: 5 and 5μL; deionized milli-Q water: 30 and 11.17μL; TEMED: 0.1 and 0.1μL; 10% APS: 1 and 1μL. The premix solutions were degassed and stored at 4°C before use.

Micro- and nano-contact printing

Using the micro- and nano-stamps, we first printed α-collagen-1 Ab patterns onto the "intermediate" surface (Tang et al., 2012), which then were cross-linked to polymerizing PAA gels by their biotin tags to streptavidin-conjugated polyacrylamide (Streptavidin-acrylamide, Thermo Fisher). For that 7-10μL of PAA was polymerized in the "sandwich" fashion between an "intermediate" patterned surface and glass-bottom 35mm Petri dishes (MatTek Corp., Ashland, MA), activated with 3-(trimethoxysilyl)propyl methacrylate (Sigma-Aldrich) in ethyl alcohol (Pharmco-Aaper) and acetic acid (Fisher Chemical) as per the commercial protocol. 3-(trimethoxysilyl)propyl methacrylate-functionalized glass surface establishes covalent bonds with the PAA gel upon its curing. Polymerized PAA "sandwiches" then were subjected to hypotonic reversible swelling in deionized water (overnight) for a gentle coverglass release from PAA gel. The resultant fluorescent PAA-nanopatterns of α-collagen-1 were incubated overnight with 1mg/mL rat monomeric collagen type-I (Corning, NY) in cold PBS (GIBCO) at 4°C, rinsed, and used for experiments.

Preparation of elastic nanotextured CG cues

Similar to the nano-patterning method, nanotextures were cast from PAA gel premixes of chosen shear modulus (G') with optimizing modifications. As the nanotexture casting master mold we used texturized nano-surfaces (NanoSurface Biomedical, Seattle, WA), cut in 1×1cm squares by diamond pencil scribbling (on the reverse side of nano-surface) and precoated with biotinylated and fluorescent tag-labeled α-collagen-1 rabbit pAb (0.1mg/mL PBS solution, 4°C, overnight). Streptavidin-conjugated polyacrylamide premix of volumes not greater than 0.5 mL was degassed in a vacuum chamber or in an ultrasonication water bath for 1 hour. In order to prevent TEMED evaporation during the procedure, TEMED is added after the degassing session. 7-10μL of PAA was polymerized in the "sandwich" fashion between α-collagen-1 Ab-coated nano-surface and glass-bottom 35 mm Petri dishes (MatTek Corp.,

Ashland, MA), activated with 3-(trimethoxysilyl)propyl methacrylate (Sigma-Aldrich) in ethyl alcohol (Pharmco-Aaper) and acetic acid (Fisher Chemical) in a vacuum chamber. After PAA curing the resultant textured patterned elastic chip was placed overnight into cold deionized water for PAA reversible hypotonic "swelling." Then the casting surface was gently peeled from the polymerized PAA surface. For a better release of the sterically interactive nano-mold, hypotonically treated PAA "sandwiches" were optionally ultrasonicated in the water bath for 10 s. Prepared elastic PAA nanotextures then were incubated with 1mg/mL rat monomeric collagen type-I (Corning, USA) in cold PBS (4°C, overnight), rinsed, and used for the cell adhesion and contact guidance assays.

Cell Contact Guidance Assays

We utilized the human breast adenocarcinoma cell line MDA-MB-231 (ATCC® HTB-26) as a model system that features an epithelial-to-mesenchymal phenotype, is invasive and metastatic, and does not express E-cadherin. Similarly, the MIA-PaCa-2 (ATCC® CRL-1420) human pancreatic ductal adenocarcinoma cell line, which possesses the cardinal oncogene and tumor suppressor gene mutations frequently observed in pancreatic cancer, was also tested to examine sensing of CG cues and confirm key findings from experiments with MDA-MB-231 cells. For cell culture and experiments, cells were maintained in DMEM with 4.5 g/L D-glucose, L-glutamine,110 mg/L sodium pyruvate (Corning Cellgro®, USA) and 10% heat-inactivated FBS (HyClone®, USA). Pharmacological inhibitors were administered 5-10 minutes prior to cell interactions with contact guidance cue substrates, and were maintained throughout the course of the experiments. Similarly, control groups were pretreated and then incubated with corresponding amounts of vehicle solvent (e.g., DMSO). All drugs concentrations were optimized to the following values: (-)-Blebbistatin (Sigma-Aldrich, 50 μM), Nocodazole (AbCam, 0.1 μM), CK666 (Tocris, 50 μM), SMIFH2 (AbCam, 50μM), Taxol (Paclitaxel) (Sigma-Aldrich, 50 nM) and their mixtures with identical corresponding individual concentrations, and are consistent with concentrations utilized in numerous reports, particularly for transformed cells. Before utilization, final concentration, drug culture medium solutions were incubated for 20 minutes in a 37°C water bath to ensure that they were fully dissolved and then the solutions were filtered through a 0.22 μm Millex®GP (Millipore, Carrigtwohill, Co, Cork, Ireland). Each cell protrusion assay run was conducted over ~1 hour at 37°C in 5% CO_2. As such, cell viability during exposure to each drug was evaluated in this time domain using the TC20 Automated Cell Counter (BioRad, USA). Samples then were fixed with cold methanol for MT visualization (−20°C, 5 minutes) or cold DMEM with 4% PFA, followed by 0.1% Triton X-100 in 1% BSA PBS. F-actin was stained with fluorescent phalloidin (Alexa Fluor phalloidin conjugates, Thermo Fisher Scientific; 10 U/mL in 1% BSA PBS) after PFA fixation, or with anti-β-Actin antibody at 5 μg/mL (Sigma-Aldrich, USA) for 1 hour in 1% BSA PBS after methanol fixation. Chromatin was labeled with 1:1000 Hoechst solution (Tocris, USA), paxillin was immunostained with mouse mAb (BD Biosciences; 5 μg/mL in 1% BSA PBS, 1 hour incubation), MTs were stained with either Alexa Fluor®-conjugated rat anti-tubulin mAb or an unlabeled version of the same mAb clone YL1/2 (AbCam; 5 μg/mL in 1% BSA PBS, 1 hour incubation). All Alexa Fluor™ fluorescent secondary antibodies (Thermo Fisher) labelings were performed at their final concentration of 5μg/mL for 1 hour in 1% BSA PBS. To characterize actin architectures we evaluated ventral and dorsal SFs and TAs. We termed the predominantly ventral and dorsal and/or ventral SF phenotype as "dorsal and/or ventral SFs" and the predominantly TAs phenotype as "transverse arcs." Cell spreading/protrusion lengths and widths along and across anisotropic micro- and nano-scale CG cues were measured from end-to-end along each direction (inscribing into the rectangle).

Imaging

High resolution 2D and 3D imaging for cell morphometric analysis was performed on a Nikon TiE stand with an A1Rsi Confocal scan head, powered by NIS-Elements Confocal software (Nikon, Japan). Objectives used were PlanApo VC 20x/0.75 NA and PlanApo VC 60xWI/1.20NA and excitation was provided sequentially using 405 nm, 488 nm and 561nm lasers. Fluorescence was collected through a 1.2 AU pinhole using emission filters of 425-475nm, 500-550nm, and 570-620nm. Pixel size was adjusted to Nyquist sampling (voxel size x,y,z for the 20x objective, j,k,l for the 60x objective). Morphometric analysis was performed by utilizing the built-in "measurement" toolbox in NIS-Elements Advanced Research software (Nikon, Japan) as an integral part of the data analysis streamline "microscopy-to-measurement-to-analysis." Video-sequences were also cut, assembled and converted into movies utilizing "stacks" toolbox in ImageJ (NIH, USA). Additionally, live cell imaging microscopy experiments were performed in microclimate-controlled stage top incubator (Tokai Hit, Japan) at 37°C in 5% CO_2, utilizing PFS (perfect focus system) as an integral part of A1Rsi Confocal scan head, powered by NIS-Elements Confocal software (Nikon, Japan). Composite 2D/3D cells plus micropattern images were reconstructed and assembled using NIS-Elements AR and linear image parametric adjustments. Figures were composed using unmodified NIS-Elements AR-generated TIFF images with Adobe Illustrator CC 2017 (Adobe Systems, Inc.). Average cells and nuclei shapes and their heatmaps were produced by superpositioning of the corresponding images into the TIFF stacks and averaging them with "Image→Stacks→Z Project→Average Intensity" function in ImageJ (NIH, USA). The resultant averaged images were analyzed with "Analyze→3D Surface Plot" function of ImageJ (NIH, USA) utilizing "heat map."

Cell traction forces analysis

For tracking deformation in polyacrylamide (PAA) gels (Polio and Smith, 2014; Ray et al., 2017) during traction force microscopy (TFM) analysis, we modified patterned PAA platforms by adding well-ultrasonicated 0.2μm fluorescent nanobeads (Polysciences) into PAA solutions (1:1000 dilution) before gel polymerization. "Before" and "after" cell removal images of the PAA micropatterns were taken with live confocal laser scanning at the interface planes between cells and the adhesion ligands patterns. Cell removal was performed by adding SDS detergent (Fisher Bioreagents, USA) to the final concentration of 0.5% (w/vol). Live cell imaging

was performed in a microclimate-controlled stage top incubator (Tokai Hit, Japan) at 37°C in 5% CO_2. Bead displacements and corresponding traction forces fields were calculated using an iterative particle image velocimetry (PIV) algorithm and an unconstrained Fourier transform traction cytometry algorithm, respectively (ImageJ plugins)(Tseng et al., 2012).

Statistical Analysis

Multiple groups were compared by ANOVA, followed by the Tukey post hoc analysis. Pairwise comparisons were analyzed using a t test. Figure legends indicate which statistical test was performed for the data. Statistical analysis was performed using either KaleidaGraph 4.5.3 (Synergy Software) or Prism 7b (GraphPad Software, Inc). Sample size N for each comparison is reported in the corresponding plots (i.e., for FA size measurements "N" reflects the number of measured individual FAs across 5-10 randomly chosen cells). For overall cell morphology measurements, e.g., cell lengths, widths or averaging of the cell shapes, N represents the number of measured cells. Data are shown as mean ± s.d.

Cell Reports, Volume *25*

Supplemental Information

Microtubule-Actomyosin Mechanical Cooperation during Contact Guidance Sensing

Erdem D. Tabdanov, Vikram Puram, Alexander Zhovmer, and Paolo P. Provenzano

Supplemental Figures:

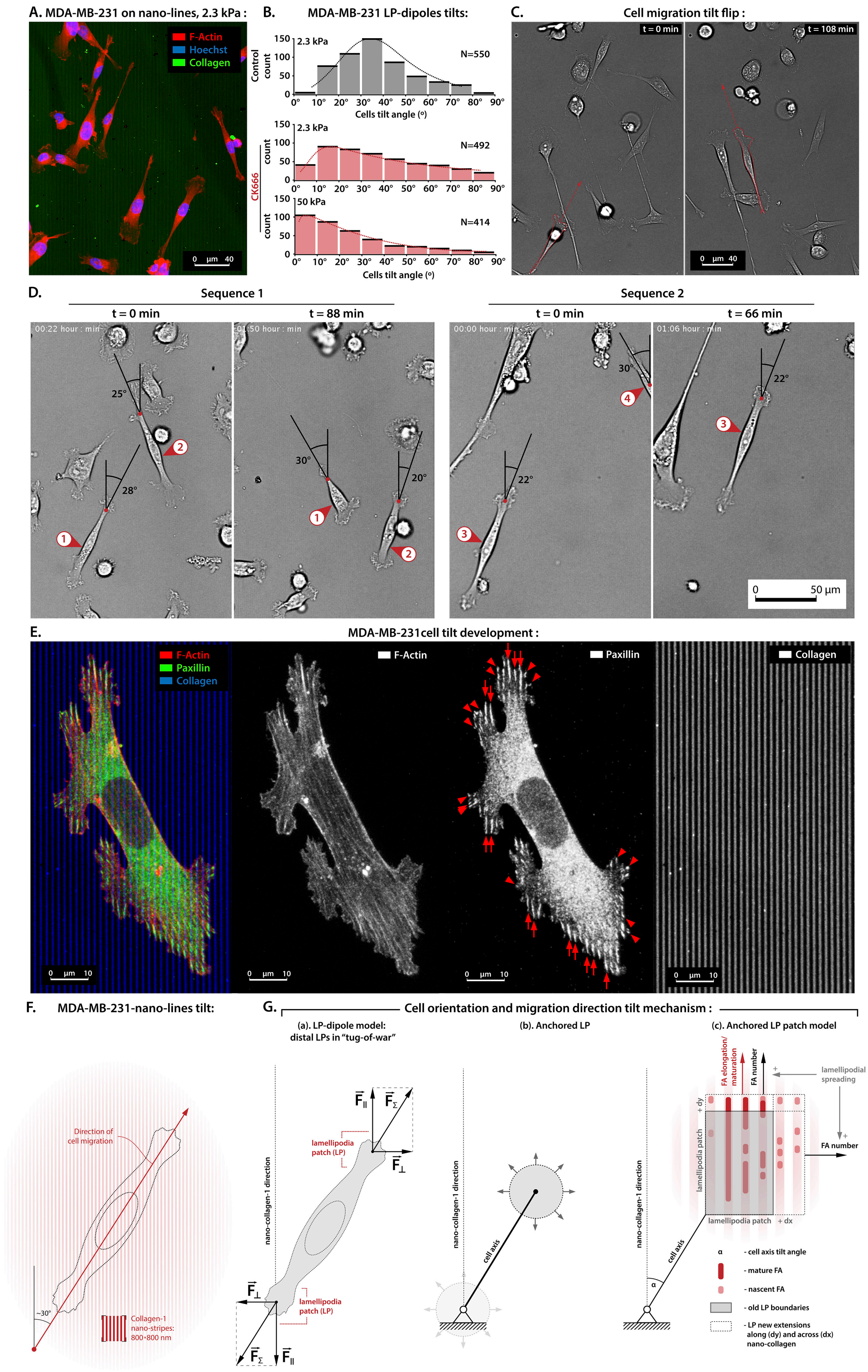

Figure S1. Related to Figures 2 and 3. Carcinoma cell axis and migration tilt relative to collagen nanoline "fibers" on compliant 2.3 kPa substrates

(A) Overview of tilting of cell populations on flat soft collagen nanolines. **(B)** Distribution of cell tilts on nanolines for the control case (+*DMSO*, 2.3kPa) and during Arp2/3 inhibition (+*CK666*, 2.3 and 50kPa collagen nanolines). Analysis of orientation of rod-shaped cells on the compliant nanolines shows the populational tilt amassing at an ~30º angle. **(C)** Time sequence of tilted cell migration capturing conservation of cell tilt during a change in cell migration direction (red outlining contour). Analysis of live cell migration dynamics indicates that the tilt is dynamically steady (see also Movie 2) and that MDA-MB-231 cells show conservation of migration tilt angle of ~30º irrespectively of of the choice between all 4 possible quadrants of cell migration directions (i.e. the migration flip or "reflection"). **(D)** Change in cell tilt tracked across two video sequences. Tilts are indicated with angles values (*black*), each flipping cell is marked with a number (*red*). **(E)** Early (t<30 min) cell elongation into LP-dipoles and its tilt (G'=2.3kPa): elongating mature FA - *red arrows*, newly established FAs - *red arrowheads*. **(F)** Schematic of a linearized cell tilted relative to the collagen nanolines. **(G)** (a) - Tilting LP-dipole cell with both $F_{||}$ - vertical forces, $F_{\perp}$ - transverse forces. (b) - LP-dipole represented as an anchored LP, (c) - Detailed schematic of LP migration tilt formation: resultant cell orientation emerges from superposition of different forces vector direction that are generated by FA maturation and elongation along the collagen nanolines, and across collagen nanolines stripes with increasing numbers of anchoring sites - nascent FAs. Thus, from this data we argue that MT-induced cell linearization into LP-dipoles on flat compliant nanolines (see Figure 2) links contractile and protrusive lamellipodial dynamics at the cell distal regions, enabling cells' slightly angled orientation along quasi-2D collagen nanolines. That is, structural and mechanical analysis of distal lamellipodii indicates a radial distribution of both FAs (Figure 2 and S2) and traction forces (Figure S2) in LPs that behave as semi-autonomous elements, but are linked via the MT-rich cell body. Thus, the rod-shaped cell phenotype represent a configuration with active dual distal LPs, interconnected into a single system (i.e. an LP-dipole entangled into a "tug-of-war" configuration that creates symmetry and principally differs from the single lamellipodia phenotype that results in more circular traction symmetry (Figure S2). Indeed, since protrusion of individual LP is primarily guided by maturation of FAs along (||) the continuous length of the CG cues and since new nascent FAs (per single lamellipodial undulation) are established as the membrane protrudes perpendicular to the nanolines (⊥), the balance of mature aligned FAs and nascent FAs protrusion establishes LP-dipole directionality that is tilted relative to the nanolines lines (panel F). Lastly, the model proposed here predicts that suppression of lamellipodial dynamics would decrease the rate and area at which the nascent FAs are established, shifting cell spreading toward the direction of mature focal adhesion elongation, hence, decreasing cell tilts. Indeed, Arp2/3 inhibition results in a decrease in lamellipodial structures at the cell ends and a significant decrease in tilt angle from ~30º to ~5-10º on both compliant and stiff nanoline (Figure 2A and S2), supporting our model. Further, analysis of the traction forces in the Arp2/3-inhibited rod-shaped cells that are devoid of LP-generated off-axis forces indicates increased force alignment along the contact guidance cues (Figure S2).

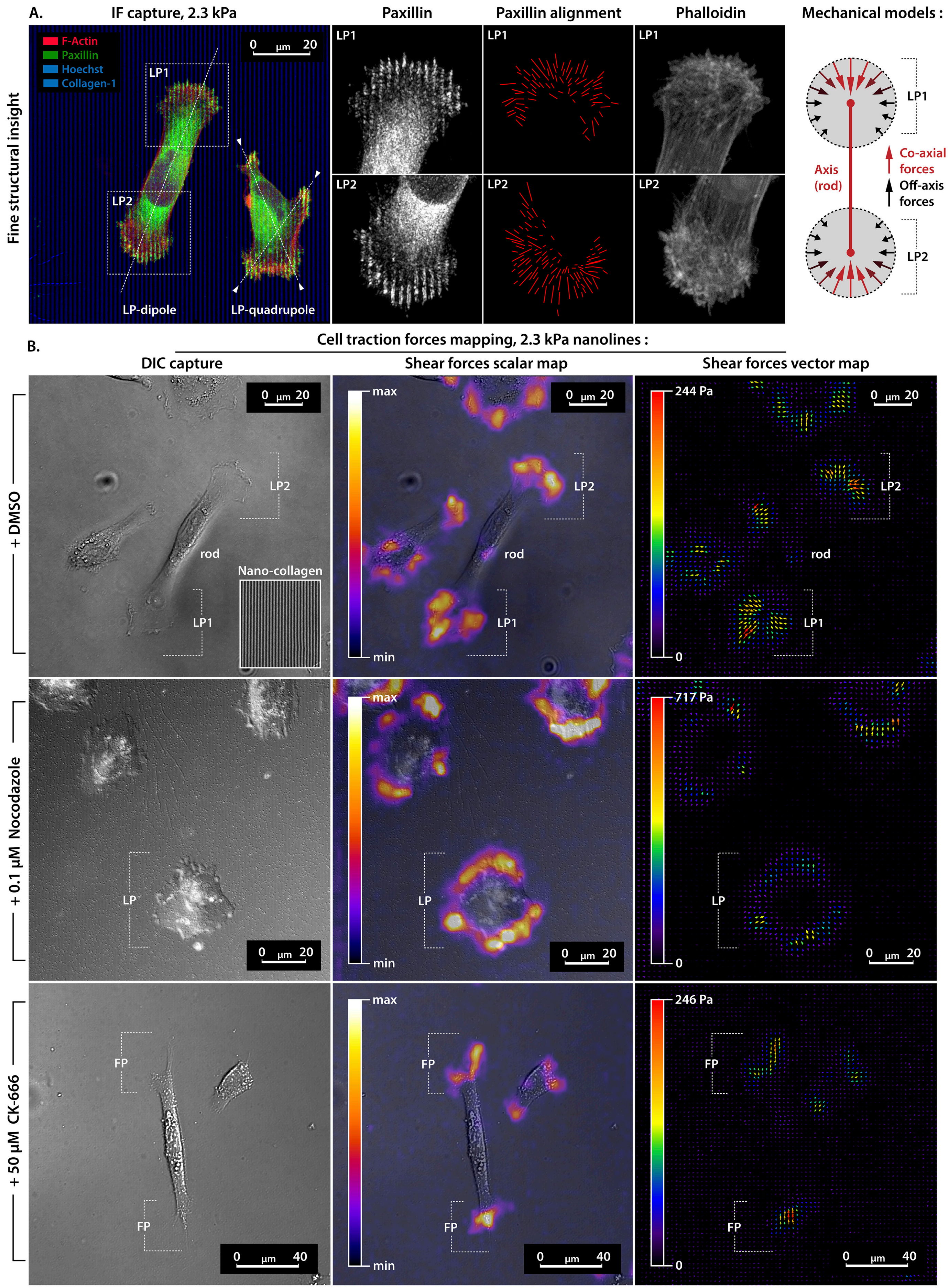

A.
IF capture, 2.3 kPa
Paxillin
Paxillin alignment
Phalloidin
Mechanical models :
Fine structural insight
F-Actin
Paxillin
Hoechst
Collagen-1
0 µm 20
LP1
LP2
LP-dipole
LP-quadrupole
Axis (rod)
Co-axial forces
Off-axis forces
B.
Cell traction forces mapping, 2.3 kPa nanolines :
DIC capture
Shear forces scalar map
Shear forces vector map
+ DMSO
+ 0.1 µM Nocodazole
+ 50 µM CK-666
rod
Nano-collagen
max
min
244 Pa
717 Pa
246 Pa
0
LP
FP
0 µm 40

Figure S2. Related to Figures 2 and 3. Analysis of cytoskeleton structure and stress alignments within distal lamellipodial patches (LP) in LP-dipoles on compliant 2.3kPa collagen nanolines
(A) Overview of cells on compliant (G'=2.3kPa) collagen nanolines substrates (*left panel*). Boxed areas with lamellipodia patches (LP1 and LP2) are magnified for detailed view (*right panels*). Note the paxillin puncta alignment (red) forms a radially organized pattern within each distal LP, outlining the partial traction autonomy of each distal LP. Simplified rod-shaped cell schematics outlines distal LPs interlinked with elongated cell body (rod). **(B)** Traction force microscopy on compliant nanolines showing the spatial distribution of traction stresses in control cells (+*DMSO*), circular cells with singular lamellipodia (+*Nocodazole*) and in rod-shaped cells with disrupted lamellipodial dynamics (+*CK666*). Note, inward-directed radially arrayed stresses in distal LPs in control rod-shaped cells (+*DMSO*). Thus, while the principal of the cell has co-axial forces communicating along the principal axis of the cell from LP1 to LP2 in an end-to-end manner, radial off-axis stresses suggest autonomous LP inward-directed contractility within each individual distal LP. MTs disruption-induced single circular lamellipodia (+*Nocodazole*) shows inward-directed peripheral stresses. Arp2/3 inhibition induces predominantly co-axial principal stresses, conforming to the observed protrusions.

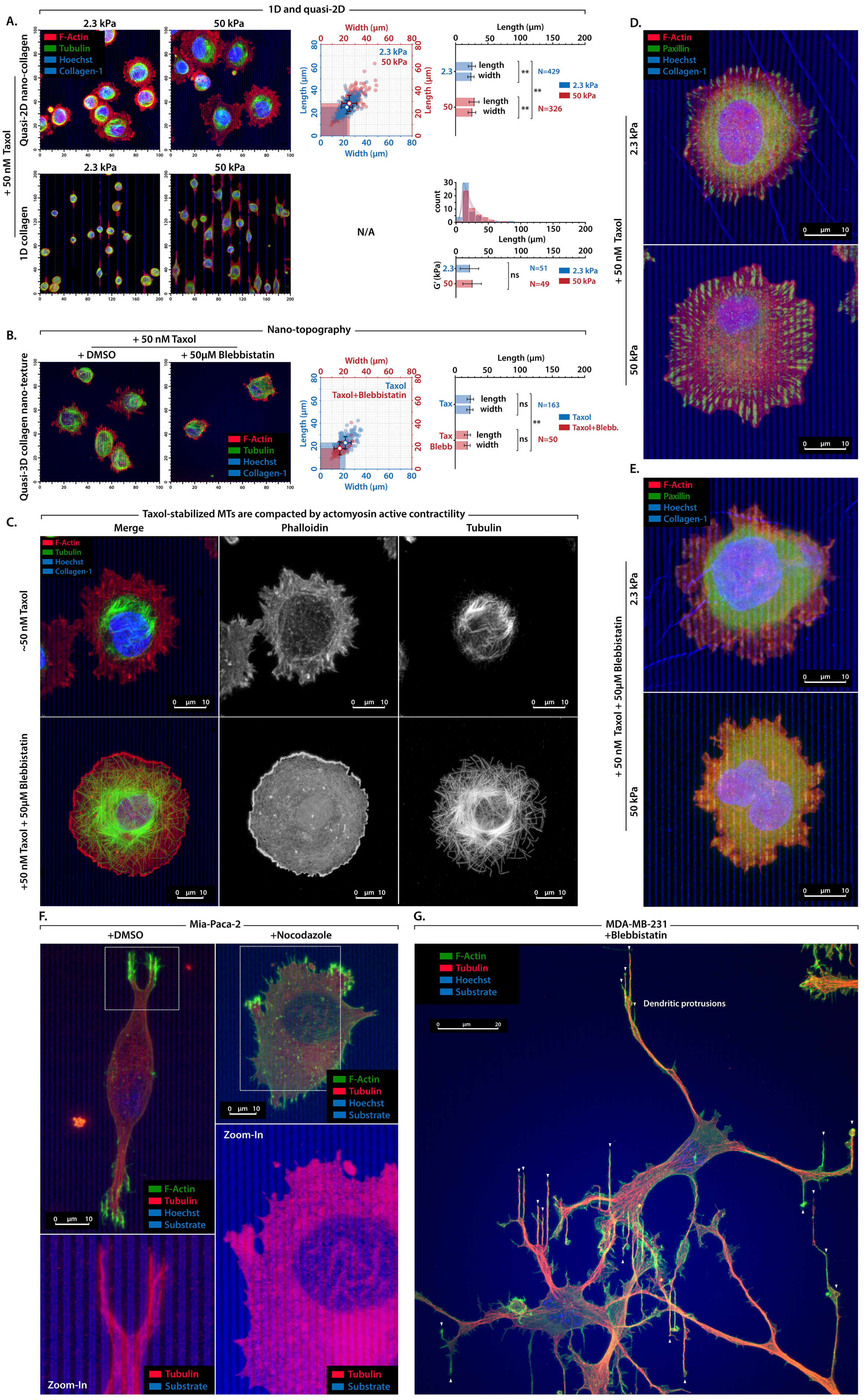

A.
1D and quasi-2D
+ 50 nM Taxol
Quasi-2D nano-collagen
1D collagen
2.3 kPa
50 kPa
F-Actin
Tubulin
Hoechst
Collagen-1
Width (µm)
Length (µm)
length
width
N=429
N=326
N/A
count
G' (kPa)
ns
N=51
N=49
B.
Nano-topography
+ 50 nM Taxol
+ DMSO
+ 50µM Blebbistatin
Quasi-3D collagen nano-texture
Taxol
Taxol+Blebbistatin
Tax
Tax Blebb
N=163
N=50
Taxol+Blebb.
C.
Taxol-stabilized MTs are compacted by actomyosin active contractility
Merge
Phalloidin
Tubulin
~50 nM Taxol
+50 nM Taxol + 50µM Blebbistatin
D.
Paxillin
E.
+ 50 nM Taxol + 50µM Blebbistatin
F.
Mia-Paca-2
+DMSO
+Nocodazole
Substrate
Zoom-In
G.
MDA-MB-231
+Blebbistatin
Dendritic protrusions

Figure S3. Related to Figures 2-5. (A-E) Taxol treatment effects on MDA-MB-231 cell alignment in response to 1D, quasi-2D, and nano-textured topographic collagen guidance cues. (F) Pancreatic ductal adenocarcinoma response to topographic collagen CG cues. (G) Blebbistatin-induced dendritic protrusions guidance by collagen nano-topographic CG cues

(A) Taxol-treated MDA-MB-231 cell elongation and spreading on compliant and stiff collagen nanolines (quasi-2D) and along 1D collagen microlines. Cell spreading widths and lengths (when applicable) are shown on the right panels. **(B)** Taxol-treated MDA-MB-231 cell spreading and elongation along and across nanotextured collagen surfaces. Cell spreading width and lengths are shown on the right panels. **(C)** Actomyosin-dependent spatial redistribution of taxol-stabilized microtubules in cells on compliant (G'=2.3kPa) nanolines. Taxol-treated MDA-MB-231 cells on compliant quasi-2D nano-collagen surfaces demonstrate isotropic (i.e. circular) spreading. In contractile cells, taxol-stabilized MTs are compacted around the nucleus while low-contractile blebbistatin-treated cells display scattered MTs distribution. **(D and E)** FA staining (paxillin) in cells on compliant (2.3kPa) and stiff (50kPa) nanolines in Taxol and Taxol+blebbistatin conditions. **(F)** Mia-Paca-2 pancreatic adenocarcinoma cell alignment to collagen nanotextures. Cells in control conditions (*+DMSO*) feature robust alignment and MT conformity to the underlying nanogrooves, consistent with findings in MDA-MB-231 breast carcinoma cells. Nocodazole-induced MT disruption disables cell alignment to the nanotexture. **(G)** Blebbistatin-induced dendritic protrusions in MDA-MB-231 cells display a partial conformity and alignment to the underlying nanogrooved textures with small MT-rich in-groove protrusions (*white arrowheads*). Data in right panels are mean ±s.d.; ns indicates no significant difference between groups; $*p<0.05$, $**p<0.001$.

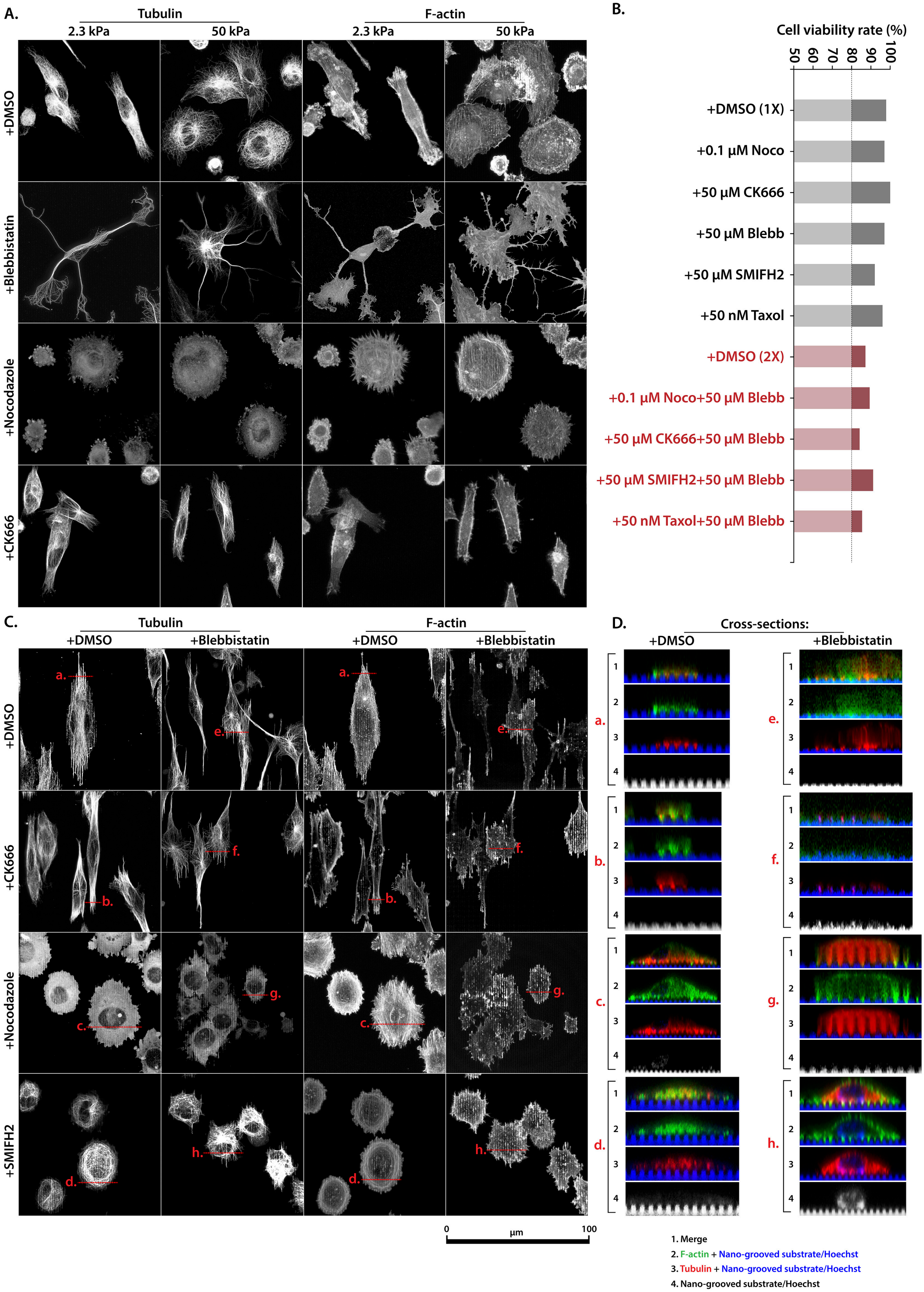

A.
Tubulin
2.3 kPa
50 kPa
F-actin
2.3 kPa
50 kPa
+DMSO
+Blebbistatin
+Nocodazole
+CK666
B.
Cell viability rate (%)
50 60 70 80 90 100
+DMSO (1X)
+0.1 µM Noco
+50 µM CK666
+50 µM Blebb
+50 µM SMIFH2
+50 nM Taxol
+DMSO (2X)
+0.1 µM Noco+50 µM Blebb
+50 µM CK666+50 µM Blebb
+50 µM SMIFH2+50 µM Blebb
+50 nM Taxol+50 µM Blebb
C.
Tubulin
+DMSO
+Blebbistatin
F-actin
+DMSO
+Blebbistatin
+DMSO
+CK666
+Nocodazole
+SMIFH2
0
µm
100
D.
Cross-sections:
+DMSO
+Blebbistatin
1. Merge
2. F-actin + Nano-grooved substrate/Hoechst
3. Tubulin + Nano-grooved substrate/Hoechst
4. Nano-grooved substrate/Hoechst

Figure S4. Related to Figures 3 and 5. (A) Tubulin and F-actin channels for cells on quasi-2D collagen nanolines depicted on Figure 3A. **(B)** Cell viability rates for pharmacological treatments outlined in Figures 3 and 5. **(C)** Tubulin and F-actin channels for cells on collagen nanotextured CG cues that are depicted in Figure 5A. **(D)** CG-transverse cross-sections of the cells depicted in Figure 5A and (C) (*corresponding cross-sections marked with red letters and dashed lines in panel C*).

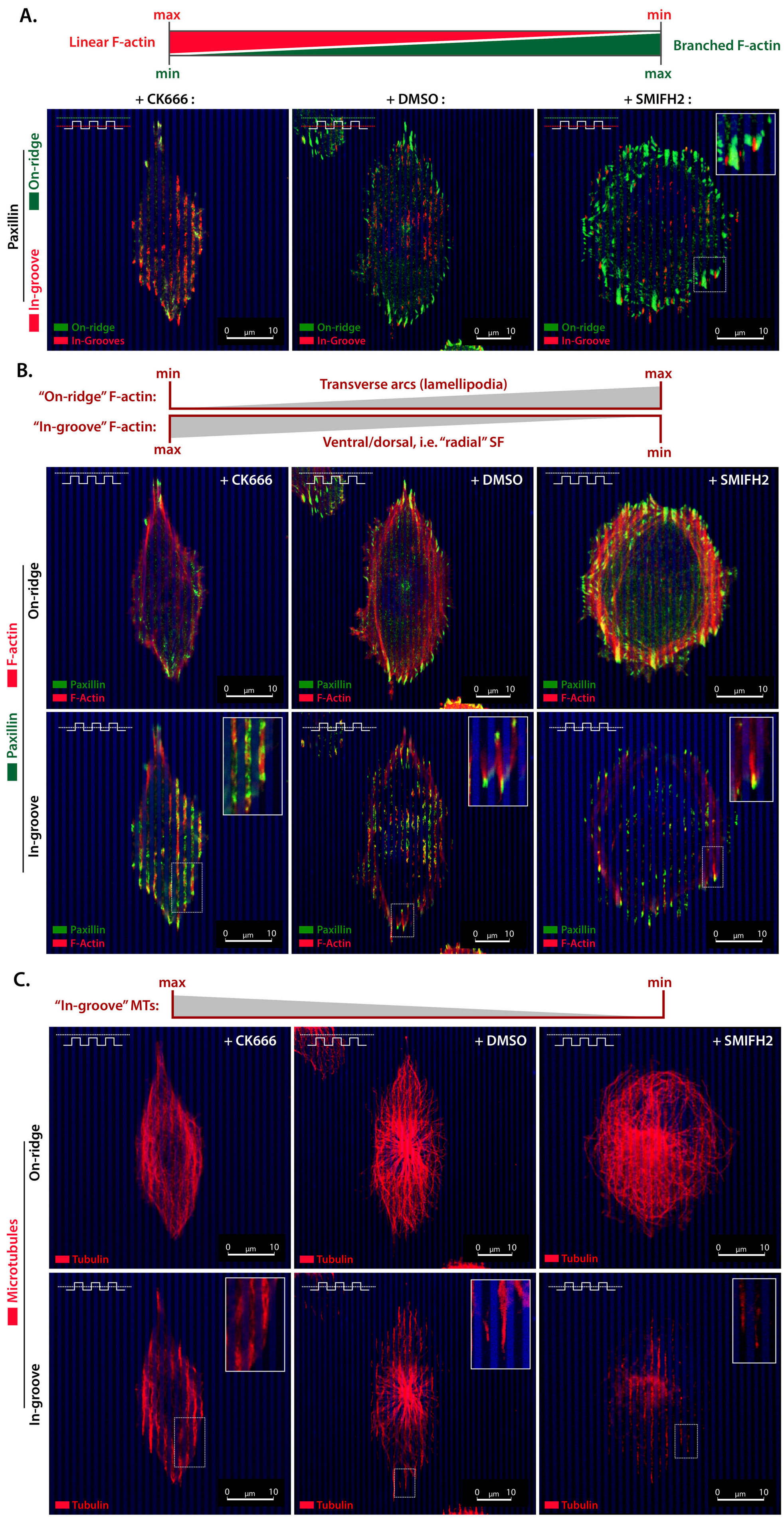
A.
max
min
Linear F-actin
Branched F-actin
min
max
+ CK666 :
+ DMSO :
+ SMIFH2 :
Paxillin
On-ridge
In-groove
On-ridge
In-Grooves
In-Groove
0 µm 10
B.
min
max
"On-ridge" F-actin:
Transverse arcs (lamellipodia)
"In-groove" F-actin:
Ventral/dorsal, i.e. "radial" SF
max
min
+ CK666
+ DMSO
+ SMIFH2
F-actin
Paxillin
On-ridge
In-groove
Paxillin
F-Actin
C.
max
min
"In-groove" MTs:
Microtubules
On-ridge
In-groove
Tubulin

Figure S5. Related to Figures 4 and 5. Apr2/3- and Formins-dependent mechanisms regulate Transverse Arcs and Ventral/Dorsal Stress Fiber F-actin architectures that regulate MT translocation between in-grooves and on-ridges layers during cellular sensing of "2.5D" collagen nanotextured CG cues.

(A) Translocation of FAs (*paxillin*) toward "in-groove" (*red*) or "on-ridge" (*green*) cell-CG interface layers during Arp2/3 (+*CK666*) or Formins (+*SMIFH2*) inhibitions, respectively. **(B)** Arp2/3 suppression (+*CK666*) diminishes on-ridge lamellipodial branched actin cytoskeleton, decreases transverse arcs, and increases robust in-groove linear F-actin structures, reminiscent to ventral/dorsal stress-fibers (*see zoomed "in-groove" inset*). Formins inhibition (+*SMIFH2*) shifts the actin architecture toward on-ridge transverse arcs and suppresses SF linear F-actin in the in-groove layer, inducing prominent F-actin translocation into on-ridge lamellipodial layer that spans atop and across multiple nanoridges. Control cells (+*DMSO*) feature both robust in-groove and on-ridge cytoskeleton architectures, representing a structural balance between Arp2/3- and Formins-regulated TA versus SF actin architectures. **(C)** Visualization of the corresponding on-ridge and in-groove MTs architectures in the cells under treatments outlined in (A) and (B). Note, decrease of the in-groove MTs as cellular F-actin transitions from predominantly linear (+*CK666*) through intermediate (+*DMSO*) to the branched, lamellipodial form (+*SMIFH2*). The measured percentage of cell-nanotexture interface areas featuring MT-positive invasive in-grooves dents for all three cases are shown on Figure 5E.
